# The NADH-dependent flavin reductase ThdF follows an ordered sequential mechanism though crystal structures reveal two FAD molecules in the active site

**DOI:** 10.1016/j.jbc.2024.108128

**Published:** 2024-12-24

**Authors:** Hendrik J. Horstmeier, Simon Bork, Marius F. Nagel, Willy Keller, Jens Sproß, Niklas Diepold, Marie Ruppel, Tilman Kottke, Hartmut H. Niemann

**Affiliations:** 1 Structural Biochemistry, Department of Chemistry, Bielefeld University, Bielefeld, Germany; 2 Industrial Organic Chemistry and Biotechnology – Mass Spectrometry, Department of Chemistry, Bielefeld University, Bielefeld, Germany; 3 Biophysical Chemistry and Diagnostics, Department of Chemistry, Bielefeld University, Bielefeld, Germany; 4 Biophysical Chemistry and Diagnostics, Medical School OWL, Bielefeld University, Bielefeld, Germany

**Keywords:** crystal structure, enzyme catalysis, enzyme kinetics, enzyme mechanism, enzyme structure, flavin adenine dinucleotide (FAD), Michaelis–Menten, nicotinamide adenine dinucleotide (NADH), X-ray crystallography

## Abstract

Two-component flavin-dependent monooxygenases are of great interest as biocatalysts for the production of pharmaceuticals and other relevant molecules, as they catalyze chemically important reactions such as hydroxylation, epoxidation, and halogenation. The monooxygenase components require a separate flavin reductase which provides the necessary reduced flavin cofactor. The tryptophan halogenase Thal from *Streptomyces albogriseolu*s is a well-characterized two-component flavin-dependent halogenase. Thal exhibits some limitations in terms of halogenation efficiency, also caused by unproductive enzyme–substrate complexes with reduced flavin adenine dinucleotide (FAD). Since the reductase components have an important regulatory function for the activity and efficiency of the monooxygenase by controlling the supply of reduced flavin, here, we studied the so far uncharacterized flavin reductase ThdF from the same gene cluster in *S. albogriseolus*, which potentially cooperates with Thal. A crystal structure of ThdF in complex with both substrates, FAD and NADH, revealed their orientation for hydride transfer. We obtained two further ThdF structures with two FAD molecules bound to the active site, suggesting a ping-pong bi-bi mechanism. In contrast, steady-state enzyme kinetics clearly showed that ThdF catalyzes flavin reduction *via* an ordered sequential mechanism, with FAD being bound first and FADH_2_ released last. Compared to related flavin reductases, ThdF has a low *k*_cat_ and low *K*_M_ value. The inhibition of ThdF by NAD^+^ might limit Thal’s halogenation activity when the cellular NADH level is low. These results provide first insights into how the efficiency of Thal could be controlled by flavin reduction at the reductase ThdF.

The regeneration of reduced flavin using NAD(P)H is an essential aspect for the function of flavin-dependent monooxygenases. While some enzymes provide the reducing activity themselves by binding both the flavin cofactor and the NAD(P)H, such as the flavin-dependent single-component Trp-halogenase AetF (1), many of these enzymes are complemented by a separate flavin reductase, forming a large variety of two-component oxidoreductase systems catalyzing a wide range of different reactions (2, 3). Often both enzymes are encoded in the same gene cluster (4).

Flavin reductases can be categorized into different classes depending on diverse factors, including sequence and structural similarity, substrate specificity, or reaction mechanism. A common classification distinguishes between class I flavin reductases, which tightly bind a prosthetic flavin in addition to the substrate flavin, and class II reductases, which lack this prosthetic flavin (5). This divergence between the two classes also manifests in the reaction mechanism. In class I reductases, the prosthetic flavin carries electrons from NAD(P)H to the oxidized substrate flavin according to a ping-pong mechanism, whereas class II reductases enable the direct electron transfer through a sequential mechanism, either ordered or disordered.

However, the overall sequence similarity among flavin reductases even inside the same reductase group is very low. In addition, there seems to be no correlation between the family classification based on sequence identity and structure and the specific type of reaction mechanism catalyzed by a flavin reductase. This is particularly the case for the HpaC-like family of short-chain flavin reductases, which are characterized by a β-barrel structure together with two conserved sequence motifs: one located in the N-terminal region ((S/T/C)xxPP) and the other in the C-terminal region (GDH) (6). Within this family, there are members that utilize a ping-pong mechanism (PheA2 (7)), as well as those that follow random (FerA (8)) or ordered sequential mechanisms (BorF (9)).

For two-component monooxygenases, the transfer of reduced flavin between the two enzymes is a central and much debated issue. In certain systems, there is striking evidence for direct protein–protein interactions that could promote channeling of the reduced flavin, preventing it from diffusing freely through the solution and reducing the probability of spontaneous reoxidation (10, 11). In other systems, transfer relies on thermodynamic control and free diffusion. In this case, the reductase generally has a greater affinity for the oxidized flavin, while the monooxygenase has a higher affinity for the reduced flavin (12).

In addition to the general principle of this transfer, its control and the regulation of flavin reduction are further important aspects for the catalytic efficiency of these systems. Different two-component monooxygenases follow different mechanisms. This includes effector-mediated regulation of reductases with an additional C-terminal autoinhibitory domain, as well as direct protein–protein interactions between reductase and oxygenase and product inhibition by oxidized pyridine dinucleotides (12). In the latter case, the reaction mechanism of the reductase might play a relevant role, since the release of reduced flavin is inhibited at high NAD(P)^+^ concentrations if NAD(P)^+^ is the first product released in an ordered sequential mechanism, as shown for the reductase ActVB (13). This indicated that physiologically, the consumption of NAD(P)H can be prevented by controlling flavin reduction at the reductase when the NAD(P)H concentration is already low. Accordingly, the activity of the oxidoreductase can be adapted to the NAD(P)H/NAD(P)^+^ ratio of the cell *via* the reductase.

In the case of flavin-dependent two-component halogenases, an additional aspect affected by the control of flavin reduction and transfer is halogenation efficiency. This is particularly true for the tryptophan 6-halogenase Thal (also called ThdH) from *Streptomyces albogriseolus*, which is involved in the biosynthesis of thienodoline (14). In the absence of the substrate tryptophan, binding of FADH_2_ to Thal results in H_2_O_2_ formation in an unproductive pathway (uncoupling) and in leakage of hypohalous acid, the active halogenating agent, from the active site (15). Under anaerobic conditions, premixing the tryptophan 7-halogenase RebH or Thal with FADH_2_ can result in the formation of catalytically unproductive flavin complexes, limiting halogenation activity (15, 16). Hence, regulation of the FADH_2_ supply by the reductase may play a critical role.

Thal is a versatile enzyme able to chlorinate and brominate l-Trp and various non-natural substrates (17, 18, 19). So far, *in vitro* halogenation with Thal employed heterologous flavin reductases like PrnF (20), BorF (21) or C1, the flavin reductase component of p-hydroxyphenylacetate 3-hydroxylase from *Acinetobacter baumannii* (15). However, recent work showed that *in vitro* not all flavin-dependent halogenases function equally well with all flavin reductases (22). The gene cluster containing the *thal* gene also encodes the flavin reductase ThdF (14). Hence, ThdF might provide reduced FADH_2_ for Thal in *S. albogriseolus*. Of note, the thienodoline biosynthetic gene cluster also encodes other two-component monooxygenases that might rely on ThdF for cofactor regeneration (14). We got interested in ThdF because crystal structures had shown that Thal can bind flavin adenine dinucleotide (FAD) only *via* its adenosine moiety, while the oxidized isoalloxazine is solvent-exposed and flexible (23). Thus, one can envisage a functional ternary complex, in which the halogenase Thal and its cognate flavin reductase ThdF are bridged by FAD. Such a complex could only form if ThdF binds FAD merely *via* its riboflavin or flavin mononucleotide (FMN) moiety but leaves the AMP moiety accessible. So far, ThdF has been characterized neither structurally nor biochemically.

In this study, we investigated ThdF by crystallography and obtained structures in complex with two FAD molecules as well as with both FAD and NADH. Steady-state kinetics revealed an ordered sequential mechanism with FAD as the first substrate to bind and FADH_2_ as the last product to exit.

## Results

### Crystal structures show that ThdF can accommodate two FAD molecules in the active site

Crystallization trials with ThdF yielded crystals diffracting moderately up to a resolution of 2.9 Å in several conditions. In all cases, the crystals clearly had a yellow color, indicating a high occupancy with FAD. We solved this structure as described in experimental procedures, but we neither fully refined the structure nor deposited it in the Protein Data Bank (PDB). In order to achieve a higher resolution through denser crystal packing, the C terminus, which was missing in the solved structure and is unstructured according to AlphaFold2 (Fig. S1), should be removed. A similar approach led to significantly increased crystallizability of the halogenase AetF (24). Therefore, a truncated version was generated *via* mutagenesis PCR using primers excluding the sequence coding for amino acids 169–193. We expressed the resulting ThdF_1−168_ and attempted to purify it. Unfortunately, this truncated variant was insoluble and could not be produced in the required quantities. As an alternative approach, the full-length protein was mixed with different proteases (chymotrypsin, trypsin, or elastase) to cleave off unstructured parts of the protein and was screened again (25, 26). The crystallization trials of ThdF in the presence of elastase resulted in two well-diffracting crystal forms (space groups: form 1: *P*2_1_2_1_2_1_; form 2: *P*2_1_) of different packing with a resolution of 1.14 Å and 1.31 Å (Table S1). We analyzed the kinetics of ThdF digestion with elastase (Fig. S2). Within the first few hours, a slightly truncated cleavage product is formed, which is shortened further to a fragment of about 18 kDa. This fragment appears to be stable and dominates on the first and second day of digestion. Mass spectrometric analysis at two different time points, each dominated by one of the major cleavage products described, revealed the sequence of the major and several minor cleavage products (Fig. S3; Table S4). The earlier/heavier cleavage product was mainly cleaved after M1 at the N terminus and after A188 at the C terminus. The later/lighter cleavage product was cleaved after A5 at the N terminus and after A171 at the C terminus. The sequences of the main species were further verified by *top-down* tandem mass spectrometry (MS/MS) analysis (Fig. S4). Since larger, well-diffracting crystals appeared several days after setting up the crystallization trials, we consider the shorter fragment to be the one that facilitates crystallization.

**Figure 1:**
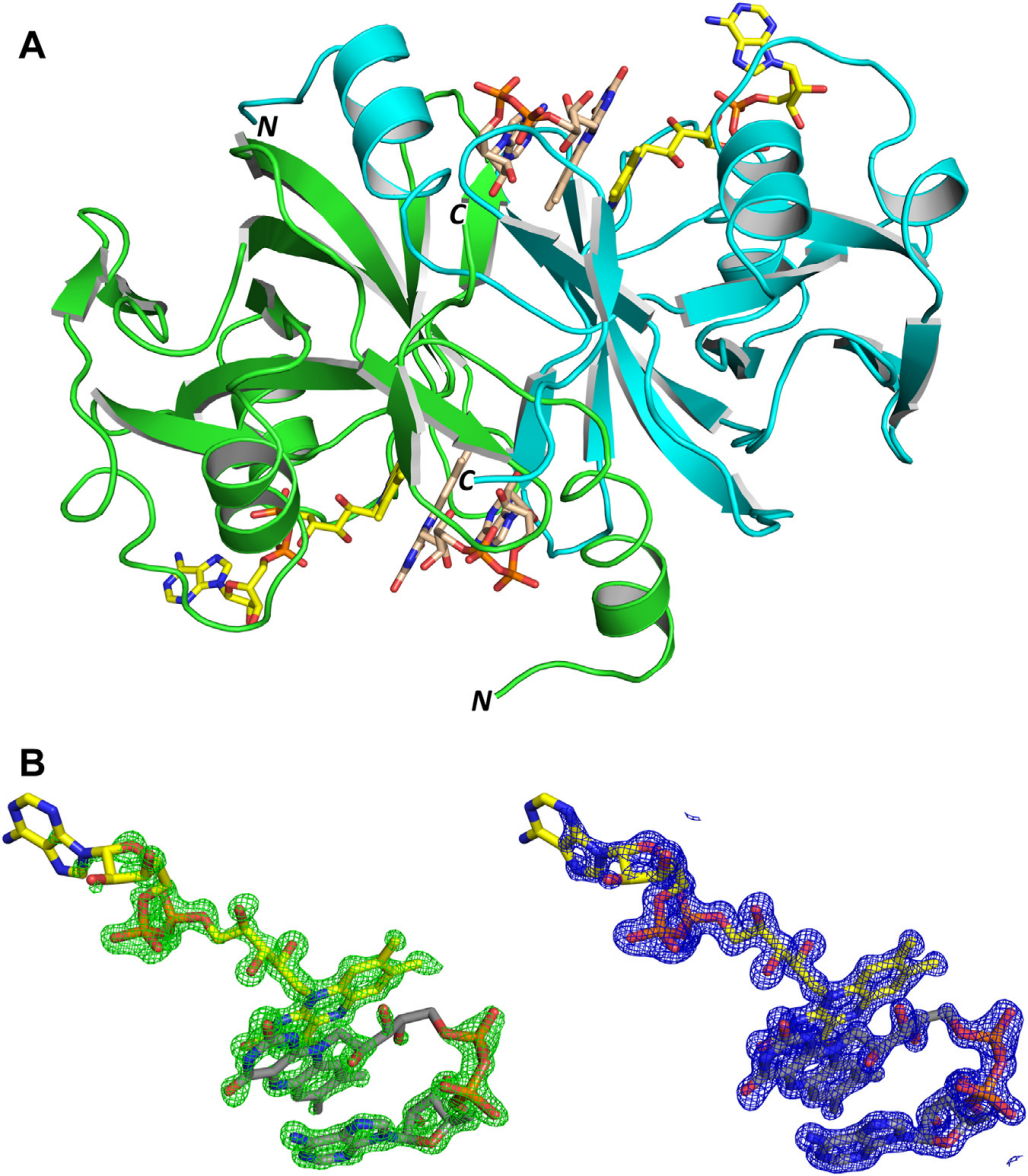
**Structure of ThdF with two FAD molecules in the active site.***A*, overall structure of the ThdF dimer as found in crystal form 1 shown as *cartoon* (chain A in *green*, chain B in *cyan*) viewing along the noncrystallographic 2-fold axis with marked N terminus and C terminus. Two FAD molecules (stretched FAD: *yellow carbon atoms*, stacked FAD: *wheat carbon atoms*) are bound per chain. *B*, electron density of bound FADs in chain A of crystal form 1. Initial *mF*_o_-*DF*_c_ density before placing both FAD molecules is shown as *green mesh* at 3σ (*left*) and final 2*mF*_o_-*DF*_c_ density is shown as *blue mesh* at 1.5σ (*right*). FAD, flavin adenine dinucleotide.

Both crystal forms contain four ThdF molecules per asymmetric unit. In all solved structures, ThdF is present as an intertwined homodimer (shown for crystal form 1 in Fig. 1*A*), which corresponds to the observations from size-exclusion chromatography (SEC). In all four chains of both crystal forms, the structure could be built completely from residue 7 (±1) – 167 (±1). The missing eight amino acids at the N terminus (including residual amino acids from tobacco etch virus (TEV) protease digestion) and 26 amino acids at the C terminus could be present but disordered or, as shown by mass spectrometry (MS) of elastase-digested ThdF, probably cleaved off by the protease in the crystallization drop. At least a major part of the C terminus has to be truncated, since the entire C terminus would not fit in the crystal packing in both crystal forms. The overall structure of ThdF does not differ between the two crystal forms (RMSD of 0.224 Å for chain A C_α_ atoms). The fold is very similar to other flavin reductases of the short-chain flavin reductase family, for example, PheA2 with RMSD of 0.701 Å for chain A C_α_ atoms (PDB 1rz0 (27)) and HpaC with RMSD of 1.136 Å for chain A C_α_ atoms (PDB 2d36 (28)). Surprisingly, in both crystal forms, two FADs with very well-defined electron density were visible per ThdF molecule (Fig. 1*B*). Based on UV-visible spectra (see below), a ratio of about 1:1 was expected here. We assume that parts of the noncrystallized protein released the bound FAD, either due to a rather weak binding or because the protein precipitated under the low salt condition used for crystallization (see below). Having two FADs bound to the active site of a short-chain flavin reductase is unusual. Of the twenty top hits from a DALI (29) search of the PDB90, only CtcQ (PDB 8ct0) had two flavin nucleotides bound to the active site, even when taking into account other structures of the same protein that are excluded from the PDB90. The active sites of the remaining 19 proteins were either empty or had one flavin nucleotide (FMN or FAD) or one flavin and one pyridine nucleotide (NAD(H) or NADP(H)) bound. As of now, there is no publication accompanying the CtcQ structure.

**Figure 2:**
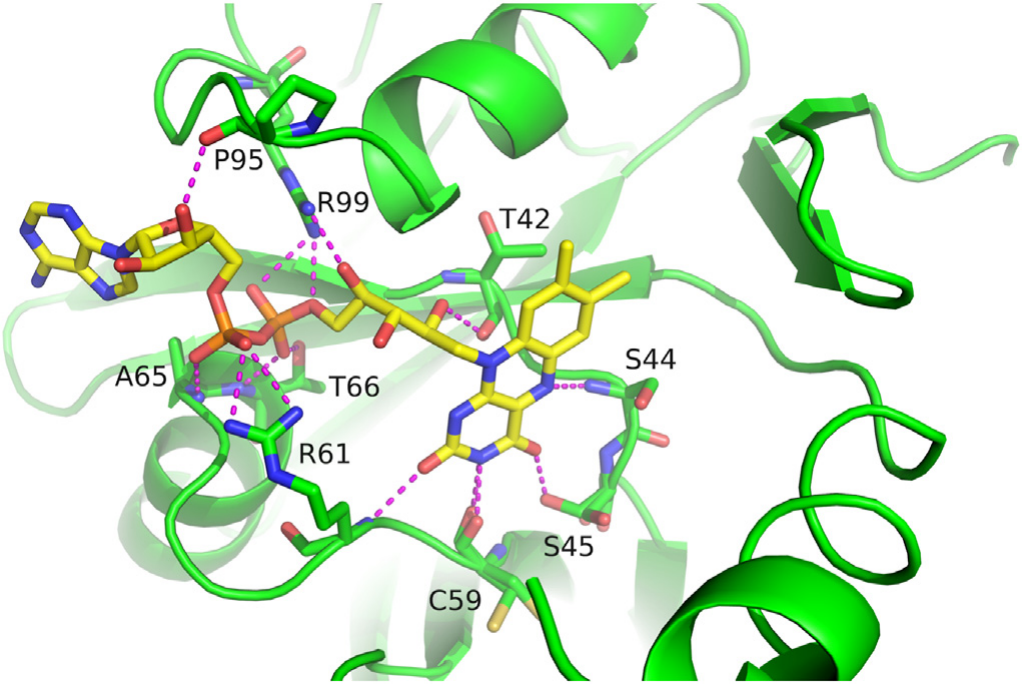
**Interactions of ThdF with the stretched FAD.** Binding of the stretched FAD (*yellow carbon atoms*) to chain A (*green cartoon*) of FAD:FAD:ThdF in crystal form 1. Direct polar contacts are indicated as *magenta dotted lines*. Only four of 14 polar contacts are formed by the AMP part of FAD and the remaining ten contacts by the FMN part. FAD, flavin adenine dinucleotide.

**Figure 3:**
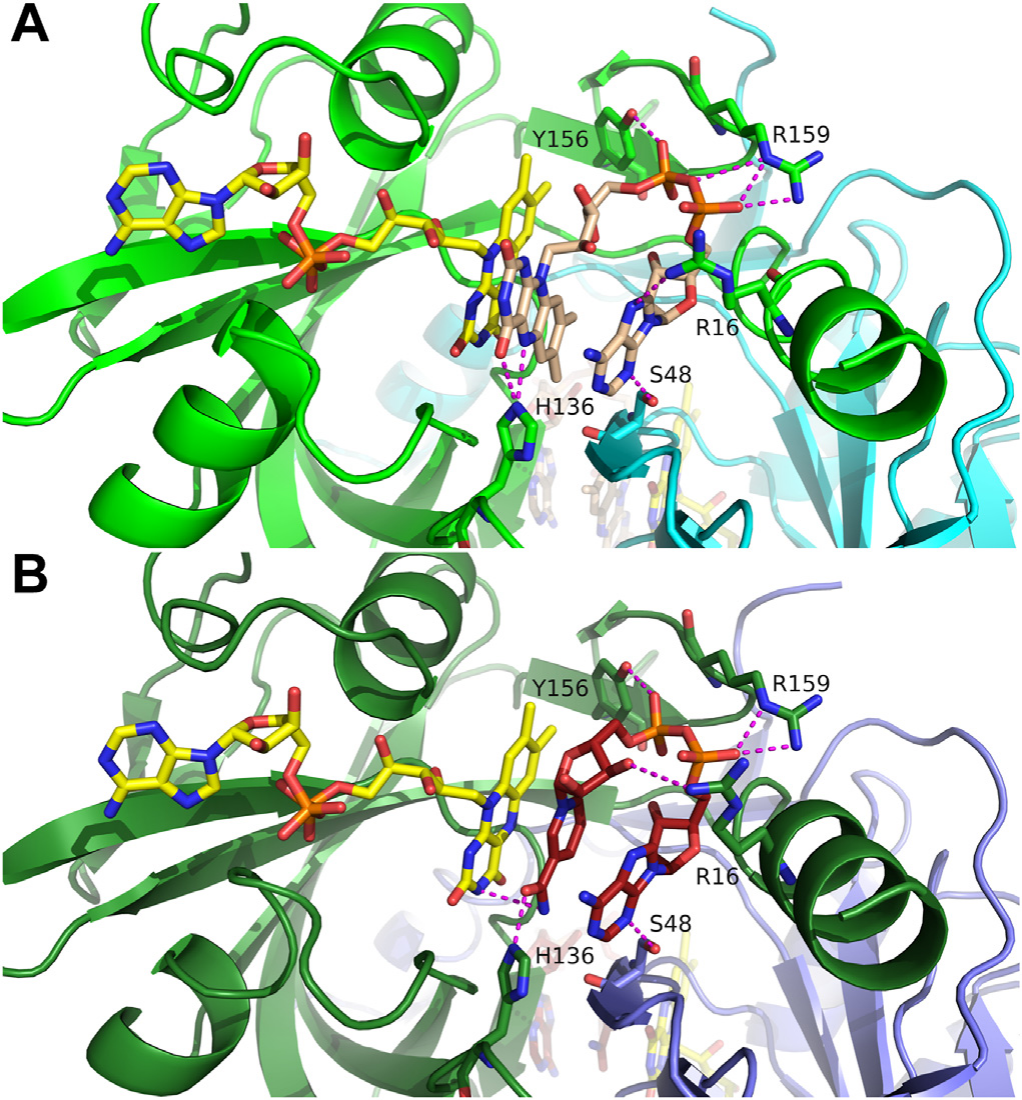
**Binding of stacked FAD and NADH in ThdF.***A*, ThdF dimer of FAD:FAD:ThdF in crystal form 1 (*green*: chain A, *cyan*: chain B) shown with bound FADs (stretched FAD: *yellow carbon atoms*, stacked FAD: *wheat carbon atoms*) of chain A. For the stacked FAD, direct polar contacts are shown as *magenta dotted lines*. Most contacts are formed to amino acid side chains of chain A of the dimer except for one contact to Ser48 of the second chain of the dimer. *B*, binding of FAD (*yellow carbon atoms*) and NADH (*red carbon atoms*) to chain A (*dark green*) and chain B (*blue*) of the NADH:FAD:ThdF dimer. NADH adopts a very similar binding pose as the stacked FAD in Figure 3*A*, also forming one polar contact (*magenta dotted lines*) to the second protomer of the ThdF dimer. FAD, flavin adenine dinucleotide.

The FAD closer to the dimer interface is stacked, bringing the adenine base and the isoalloxazine ring into close proximity. The peripheral FAD is extended. The stacked FAD is completely enveloped by 2*mF*_o_-*DF*_c_ density in all chains, while in some chains, the adenosine moiety and especially the adenine of the stretched FAD has poor or even no electron density when contoured at 1.5σ (Fig. 1*B*). This is consistent with the interactions with surrounding amino acids. The two bound flavins have a large number of direct interactions with surrounding amino acids. The stretched flavin forms 14 polar contacts to surrounding amino acids (Fig. 2). Ten of these contacts are from the FMN part of the FAD. Conserved polar contacts with the side chain of R61 and the backbone NH of A65 fix the phosphate from AMP in a defined position. The adenosine has only one polar contact to the protein involving the ribose. This contact differs between chains. The adenosine is flexible and adopts a flipped conformation in chain A of crystal form 1 (Fig. S5), suggesting that FMN should be able to bind almost as well as FAD. The stacked FAD forms eight polar contacts to surrounding amino acids (Fig. 3*A*). Since the binding of two FADs is necessary for flavin reductases that function *via* a ping-pong mechanism, the structures suggest such a mechanism. It is noteworthy that the orientation of the isoalloxazine rings differs from the only structures of known ping-pong flavin reductases in complex with two flavins that we found in the PDB, namely, EmoB (PDB 4ltm (30)) and TTHA0420 (PDB 1yoa (31)). In the case of EmoB, the long axes of the isoalloxazines of two bound FMNs are antiparallel to each other, whereas in ThdF, they are rotated by approximately 90° (see below). While the planes of the isoalloxazine moieties of both FADs are almost parallel in ThdF and EmoB, they form a tilt angle of roughly 45° in TTHA0420.

In order to determine at which binding site the cofactor NADH binds and to investigate whether the structures with two FADs are a functional state, we aimed at solving an additional structure in complex with NADH. However, all soaking and cocrystallization experiments with NADH at different concentrations resulted in structures with two bound FADs. Only by soaking with NADH and subsequent addition of dithionite, which led to a clearly visible decolorization of the crystals (Fig. S6), we obtained a structure in crystal form 1 (*P*2_1_2_1_2_1_) with bound NADH.

**Figure 4:**
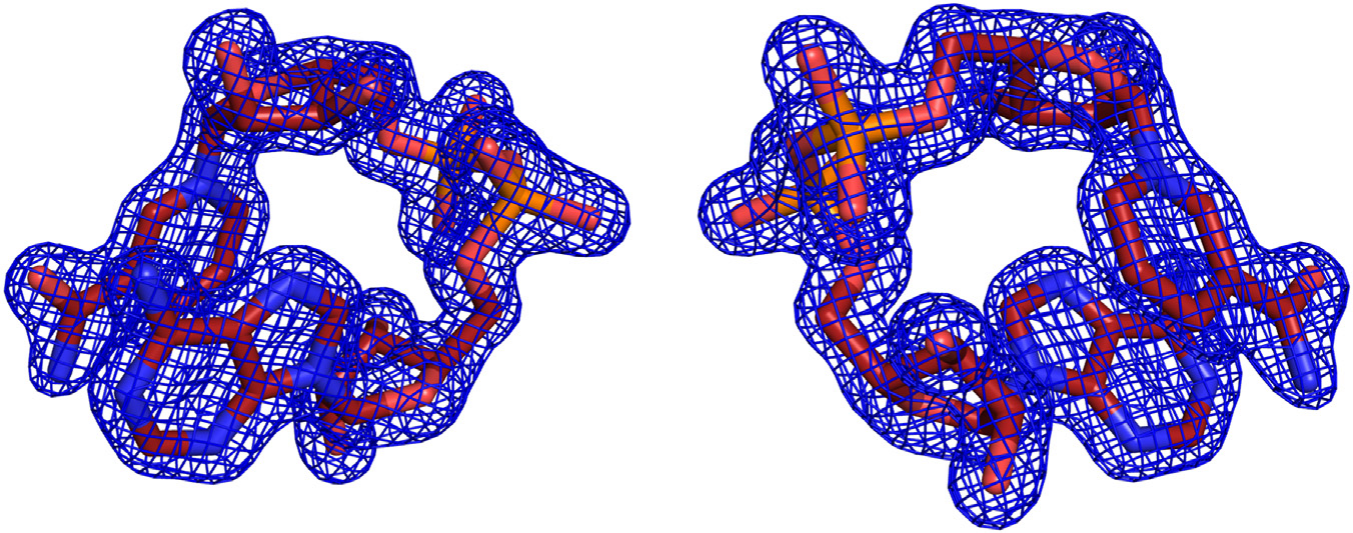
**Electron density of bound NADH.** The final 2*mF*_o_-*DF*_c_ density is shown as *blue mesh* at 1.5σ in two views (rotated by 180°).

In the NADH:FAD:ThdF complex, the NADH binds instead of the stacked FAD and in a very similar way (Fig. 3*B*). The electron density for NADH is well defined (Fig. 4). The AMP part of NADH adopts a binding pose almost congruent to that of the stacked FAD. Accordingly, both nucleotides form the same polar contacts to surrounding amino acids. Only the side chain of R16 adopts a different rotamer and thus forms a polar contact to the ribose of the nicotinamide mononucleotide (NMN) part in the NADH instead of the adenosine in the FAD. H136, which is part of the conserved GDH motif, binds the isoalloxazine ring of the stacked FAD. When NADH is bound, H136 forms a polar contact to the nicotinamide instead, presumably positioning the two reactive heterocycles of the stretched FAD and NADH for efficient hydride transfer. Despite the clear decolorization of the crystals after the addition of dithionite, the electron density of the isoalloxazine moiety of the stretched FAD is planar. Bending of the isoalloxazine would have been a clear indicator for a reduced state, but it is important to note that reduced flavin can still appear planar (32). Both centrally bound ligands—the NADH and the stacked FAD—form a polar contact to the side chain of S48 of the second protomer of the ThdF dimer. Binding of a nucleotide at this site might be important to stabilize the ThdF dimer.

### In solution, ThdF can bind one or two FAD molecules

**Figure 5:**
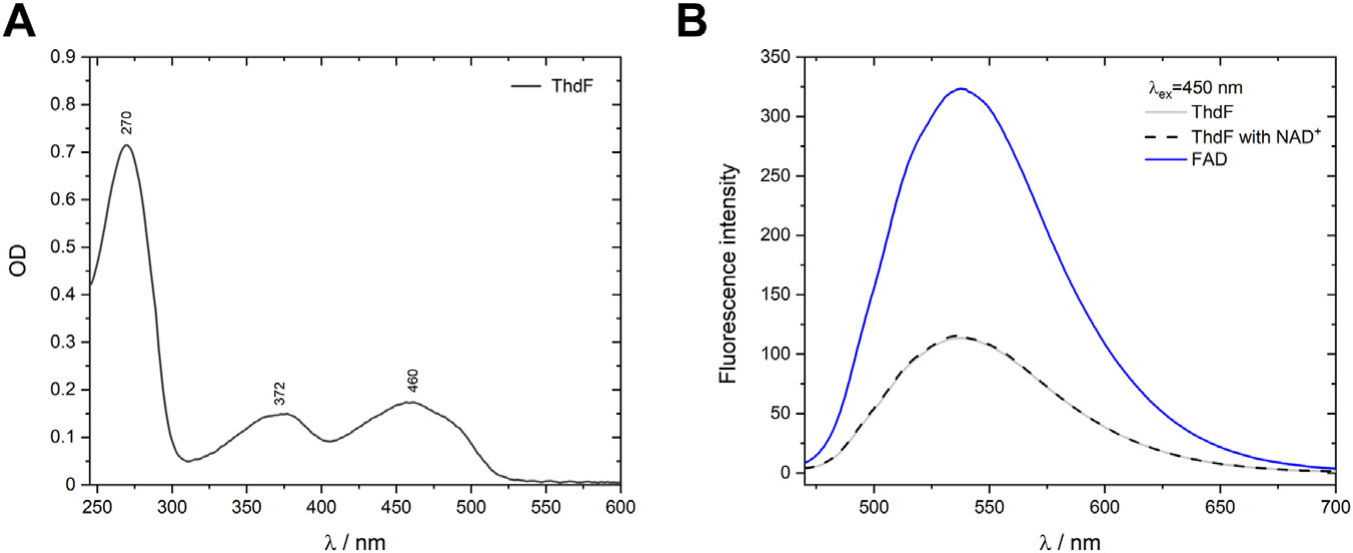
**Spectroscopic investigation of ThdF in solution.***A*, UV-visible spectrum of ThdF (12.5 μM). Calculation of the cofactor occupancy yielded a value of 120%. *B*, fluorescence emission spectra after excitation at 450 nm of (i) purified ThdF with one bound FAD (occupancy of 120%), (ii) ThdF with 1 mM NAD^+^, and (iii) FAD. Fluorescence of FAD bound to ThdF is quenched compared to free FAD and remains unchanged in the presence of NAD^+^, indicating that in solution the stretched FAD from the crystal structure is present. FAD, flavin adenine dinucleotide.

To clarify whether the ThdF structure with two FAD molecules per monomer could represent a functionally relevant state, we investigated the cofactor occupancy of ThdF (same batch as for crystallization) in solution by UV-visible spectroscopy (Fig. 5*A*). The spectrum showed the typical signals of a flavoprotein with absorption maxima close to 372 and 460 nm. We calculated the ThdF:FAD ratio from absorption at 280 nm and 450 nm. To determine the FAD concentration using the published extinction coefficient of FAD at 450 nm, we removed the protein by boiling and centrifugation because protein binding changes the absorption spectrum of FAD (Fig. S7). The mean FAD occupancy of ThdF from three independent purifications was 124 ± 4%. The anaerobic reduction of FAD in ThdF by dithionite supports a partial occupancy with two FADs in solution (Fig. S8). In the course of the reduction to the fully reduced state, an FAD neutral radical is formed with a maximal concentration of 14% relative to the initial concentration of oxidized FAD. This finding indicates that a minor fraction of the protein stabilizes the radical, most likely the fraction with two FAD bound.

To investigate which of the two binding sites might be more prominently occupied, we recorded fluorescence spectra of free FAD and of ThdF, which showed a significant quenching of the fluorescence of ThdF-bound FAD compared to free FAD (Fig. 5*B*). From the ratio of the fluorescence intensity of FAD in the free and in the ThdF-bound state, a quenching factor (Q) of 0.35 was determined for ThdF. We aimed to distinguish between the two possible binding sites by adding NAD^+^. Binding of NAD^+^ should displace the stacked FAD and release it into the solution, while the stretched FAD should remain unaffected. A release of FAD from the protein should be detectable by increased fluorescence. Since addition of NAD^+^ in excess did not change the fluorescence intensity, this suggests that FAD mainly occupies the canonical FAD binding site of flavin reductases corresponding to the stretched conformation in the crystal structure.

In addition, native protein MS revealed the existence of a ThdF dimer with four bound FAD (ThdF_2_FAD_4_) as the major species in solution, among other ratios (ThdF_2_FAD_3_, ThdF_2_FAD_2_, and ThdF_2_FAD_1_) (Fig. S9). This demonstrates that in solution both binding sites can actually be occupied. However, the dominance of the ThdF_2_FAD_4_ species might at least in part be due to the nonphysiological, almost salt-free buffer (50 mM ammonium acetate) required for electrospray ionization (ESI) MS. The melting temperature of apoThdF and ThdF:FAD determined by differential scanning fluorimetry analysis showed that FAD has a stabilizing effect on ThdF, which is reduced with increasing salt concentration (Fig. S10). This suggests that the salt concentration indeed influences binding of FAD to ThdF, which complicates the understanding of what exactly happens under nonphysiological salt-free conditions used in native ESI MS and for crystallization.

### Steady-state kinetics reveals an ordered sequential reaction mechanism

**Figure 6:**
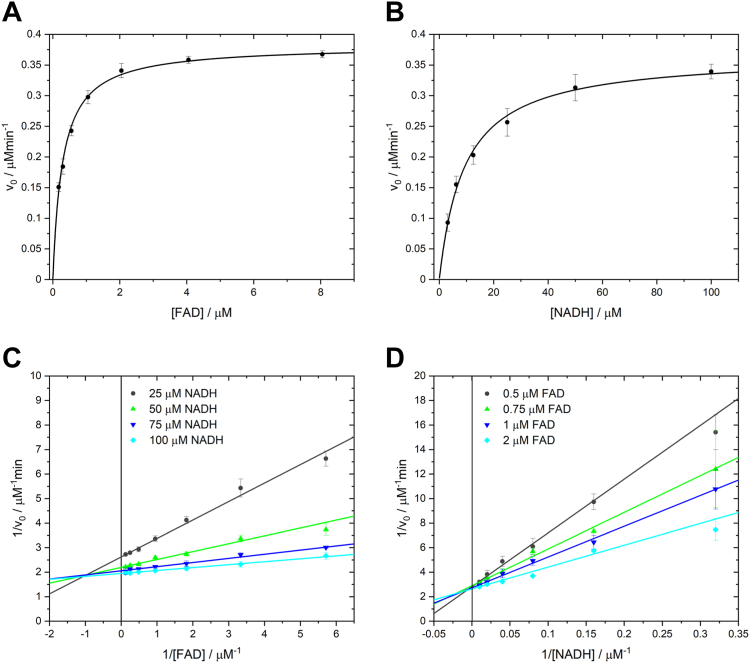
**Steady-state initial velocity kinetics of ThdF.***A*, initial velocities of ThdF at 25 μM NADH and 0.1 to 8 μM FAD. *B*, initial velocities of ThdF at 1 μM FAD and 3.1 to 100 μM NADH. *C*, FAD-dependent measurement at different fixed NADH concentrations, shown as double reciprocal plot of initial velocity *versus* FAD concentration. *Lines* show an intersecting pattern with the intersection point on the *left side* of the ordinate. *D*, NADH-dependent measurement at different fixed FAD concentrations, shown as double reciprocal plot of initial velocity *versus* NADH concentration. *Lines* show an intersecting pattern with the intersection point on the ordinate. Data points represent the mean and SD of three technical replicates on one plate. Each entire experiment was performed three times independently on different days (data not shown). FAD, flavin adenine dinucleotide.

Based on the partial double occupation with FAD molecule to ThdF in solution and the crystal structure with two FAD molecules per ThdF protomer, the question arose by which mechanism ThdF catalyzes electron transfer. The tight binding of one FAD in solution and the crystallographic observation that one of the two binding pockets can bind both FAD and NADH hints to a ping-pong mechanism. In contrast, the relative orientation of the two isoalloxazine rings of neighboring FAD molecules does not appear optimal for hydride transfer. To clarify this question, we used steady-state kinetics to elucidate the reaction mechanism of ThdF. We determined the initial reaction rates as a function of the concentration of one substrate at a fixed concentration of the second substrate and *vice versa* (Fig. 6, *A* and *B*). Performing these measurements for different concentrations of the fixed substrate resulted in intersecting lines in both cases when plotting the inverse of the initial rate against the inverse concentration of the varied substrate (Fig. 6, *C* and *D*). This observation argues against a ping-pong mechanism for ThdF, since a ping-pong mechanism should result in parallel lines for these plots (33).

Another important observation was the exact location of the intersection point, which differed between the two substrate-dependent measurements. In the FAD-dependent experiment, the intersection was located on the left side of the ordinate (Fig. 6*C*), while in the NADH-dependent measurement, the lines intersected directly on the ordinate (Fig. 6*D*). This specific combination is consistent with the pattern of an ordered sequential mechanism with FAD as the first and NADH as the second substrate (33).

**Figure 7:**
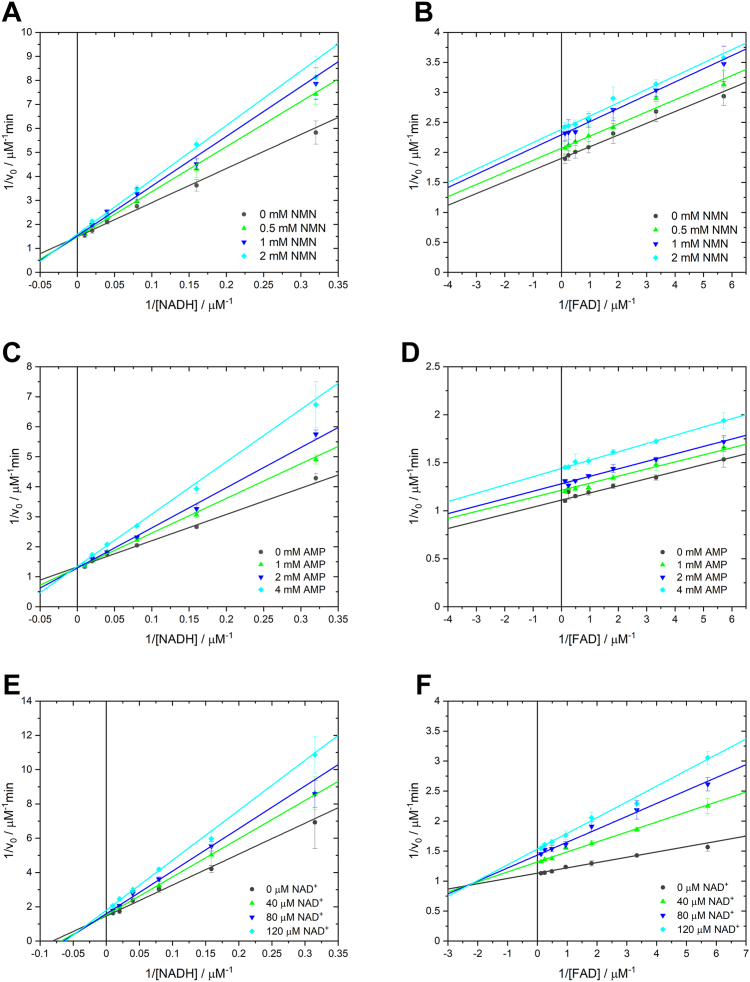
**Inhibition of ThdF with NMN, AMP, and product inhibition with NAD**^**+**^. *A*, NADH-dependent measurement at 1 μM FAD for different NMN concentrations, shown as a double reciprocal plot of initial velocity *versus* NADH concentration. The lines intersecting on the ordinate indicate competitive inhibition of NADH by NMN. *B*, FAD-dependent measurement at 80 μM NADH for different NMN concentrations, shown as double reciprocal plot of initial velocity *versus* FAD concentration. The *parallel pattern of the lines* indicates an uncompetitive inhibition pattern of FAD by NMN. *C*, NADH-dependent measurement at 1 μM FAD for different AMP concentrations, shown as a double reciprocal plot of initial velocity *versus* NADH concentration. The lines intersecting on the ordinate indicate competitive inhibition of NADH by AMP. *D*, FAD-dependent measurement at 80 μM NADH for different AMP concentrations, shown as double reciprocal plot of initial velocity *versus* FAD concentration. The *parallel pattern of the lines* indicates an uncompetitive inhibition pattern of FAD by AMP. *E*, NADH-dependent measurement at 1 μM FAD for different NAD^+^ concentrations, shown as a double reciprocal plot of initial velocity *versus* NADH concentration. The *line pattern* indicates a noncompetitive inhibition of NADH by NAD^+^. *F*, FAD-dependent measurement at 80 μM NADH for different NAD^+^ concentrations shown as double reciprocal plot of initial velocity *versus* FAD concentration. The *line pattern* indicates a noncompetitive inhibition pattern of FAD by NAD^+^. Data points represent the mean and SD of three technical replicates on one plate. Each entire experiment was performed three times independently on different days (data not shown). FAD, flavin adenine dinucleotide; NMN, nicotinamide mononucleotide.

To verify this mechanism, we performed kinetic experiments with the addition of NMN and NAD^+^ as inhibitors. The concentration of one substrate was varied with a fixed concentration of the second substrate and different concentrations of the inhibitor. We chose NMN as a potential competitive inhibitor for NADH because, in the case of an ordered mechanism with FAD as the first substrate, a competitive inhibitor for the second substrate should result in an uncompetitive-like inhibition pattern for FAD (33). The NADH-dependent measurement with NMN showed intersecting lines on the ordinate, confirming competitive inhibition toward NADH (Fig. 7*A*). The FAD-dependent assay with NMN then indeed showed a pattern of parallel lines characteristic of uncompetitive-like inhibition (Fig. 7*B*). The use of AMP instead of NMN showed the same pattern as observed for NMN for both substrates (Fig. 7, *C* and *D*). Thus, the results for inhibition of ThdF with NMN and AMP also suggest an ordered sequential mechanism with FAD as the leading substrate.

To investigate the order of product release, we performed inhibition studies with NAD^+^ as the product inhibitor. For both substrates, NAD^+^ was found to produce a noncompetitive inhibition pattern (Fig. 7, *E* and *F*), consistent with NAD^+^ being the first product released in the ordered sequential mechanism (33). Inhibition of ThdF by NAD^+^ might serve to limit Thal’s halogenation activity when NADH supply in the cell is scarce.

**Figure 8:**
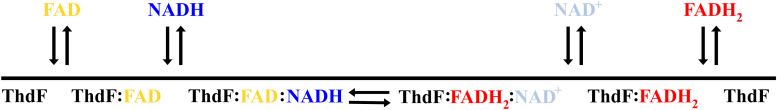
**Ordered sequential mechanism of ThdF.**

Figure 8 shows the reaction mechanism suggested by the combined kinetic data. An ordered sequential mechanism is incompatible with the presence of a tightly bound prosthetic flavin. Thus, in a first step, the extended FAD binds to nucleotide-free ThdF as the leading substrate, followed by binding of the stacked NADH as the second substrate. After direct electron transfer between NADH and the extended substrate FAD, NAD^+^ dissociates as the first product, followed by release of the reduced FADH_2_ as the second product. The stacked FAD bound in the NADH binding site in two of our structures most likely does not represent a functional state. As mentioned before, we assume that the unphysiologically low salt concentration during crystallization may favor the presumably nonphysiological state with two FADs per ThdF protomer.

### Compared to related flavin reductases, ThdF has a low k_cat_ and a low K_M_ for FAD

In addition to studying the reaction mechanism of ThdF, we also used steady-state kinetics to determine the kinetic parameters of ThdF. We performed measurements at different concentrations of FAD or NADH while keeping the second substrate at a saturating concentration and plotted the initial rates against the concentration of the varied substrate. Fitting the resulting curves with the Michaelis–Menten equation yielded the *K*_M_ and *k*_cat_ values listed in Table 1. *K*_M_ values were also determined by fitting the data with the kinetic equation for an ordered sequential mechanism (Equation S1), yielding approximately the same results. We also performed these measurements with FMN as a variable substrate, demonstrating that ThdF accepts FMN as a substrate. The *K*_M_ value for FMN does not differ from the value for FAD within the range of the measurement error. This is in good agreement with the observed crystal structure, in which the adenosine part of the FAD is less well resolved than the FMN moiety, which forms the majority of polar contacts to the surrounding amino acids. The adenosine part of the FAD is therefore not necessarily required for binding to ThdF.

**Table 1:** Kinetic parameters of ThdF

Variable substrate	Fixed substrate	Michaelis–Menten		Sequential
		*K*_M_/μM	*k*_cat_/s^−1^	*K*_M_/μM
FAD	NADH (100 μM)	0.34 ± 0.09	0.21 ± 0.08	0.34 ± 0.08
FMN	NADH (100 μM)	0.28 ± 0.07	0.20 ± 0.08	0.29 ± 0.07
NADH	FAD (10 μM)	19.5 ± 5.6	0.26 ± 0.02	10.8 ± 3.6

The values shown are the mean and standard deviation of three independent measurements performed on different days and in triplicate for each data point.

FAD, flavin adenine dinucleotide.

The *K*_M_ value for NADH (19.5 μM) is similar, that for FAD (0.34 μM) is about four to 10 times lower than other kinetically studied short-chain flavin reductases such as BorF (*K*_M, FAD_: 1.3 μM; *K*_M, NADH_: 27 μM) (9), AbeF (*K*_M, FAD_: 1.5 μM; *K*_M, NADH_: 26 μM) (9), or PrnF (*K*_M, FAD_: 3.2 μM; *K*_M, NADH_: 43 μM) (34). The *k*_cat_ value of ThdF is strikingly low with only 0.21  ± 0.08 s^−1^ (12.7  ± 4.9 min^−1^) for FAD. For comparison, the *k*_cat_ values for either substrate are around 55 s^−1^ for BorF, 100 s^−1^ for AbeF, and 65 s^−1^ in case of PrnF.

In contrast to the low enzymatic activity (*k*_*cat*_), the catalytic efficiency of ThdF is very comparable with other flavin reductases like HpaC_Tt_ and ActVB, which also catalyze ordered sequential mechanisms for flavin reduction (Table 2). However, some flavin reductases such as FerA, AbeF, BorF, and PheA2 show a higher catalytic efficiency.

**Table 2:** Comparison of the kinetic efficiency of ThdF with other flavin reductases

Enzyme	*k*_cat_/*K*_M_ /s^−1^ M^−1^	*k*_cat_/*K*_M_ /s^−1^ M^−1^	Mechanism	Reference
	*FAD variable* *NADH fixed*	*NADH variable* *FAD fixed*		
ThdF	6.2·10^5^	1.3·10^4^	Ordered sequential	This work
HpaC_Tt_	2.7·10^5^	1.9·10^4^	Ordered sequential	(55)
ActVB^a^	9.4·10^5^	1.4·10^6^	Ordered sequential	(56)
BorF	4.2·10^7^	2.1·10^6^	Ordered sequential	(9)
AbeF	6.7·10^7^	4.2·10^6^	Random sequential	(9)
FerA^b^	2.3·10^7^	1.5·10^6^	Random sequential	(8)
PheA2	1.7·10^8^	2.6·10^7^	Ping Pong	(7)

FAD, flavin adenine dinucleotide.

a For ActVB, values for NADH were determined with FMN fixed.

b For FerA, values are for FMN instead of FAD.

### The FAD affinity of ThdF supports a sequential reaction mechanism

**Figure 9:**
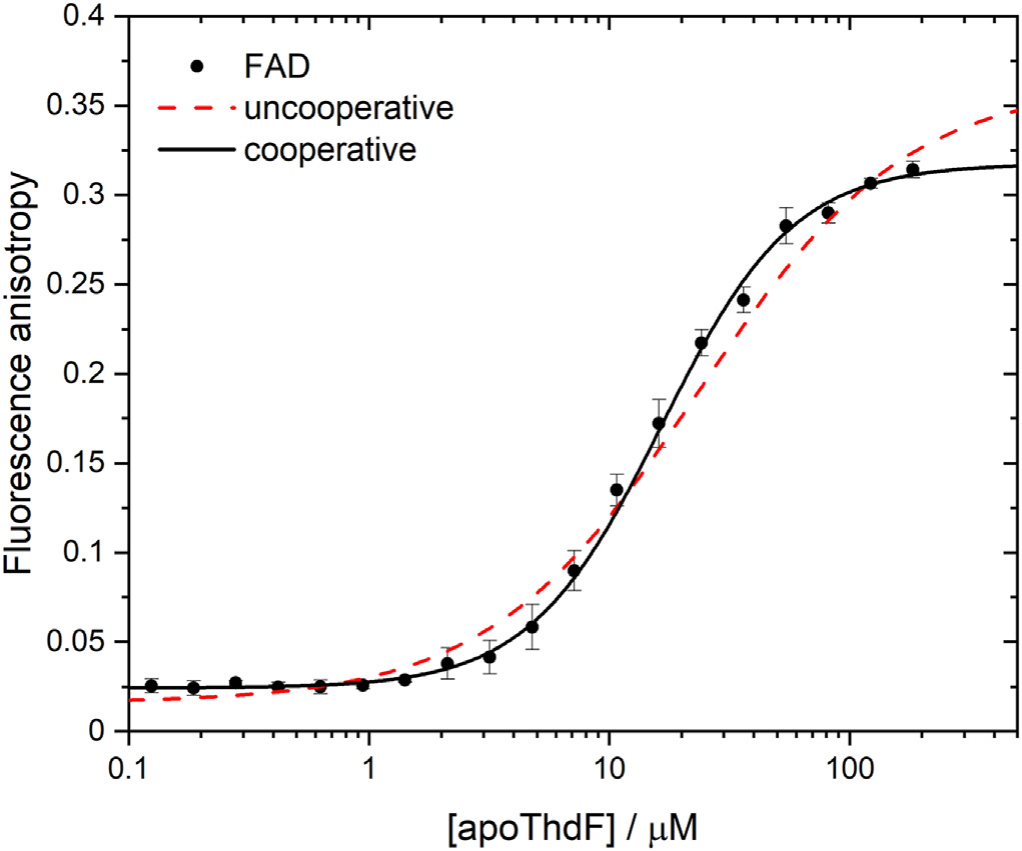
**Binding of FAD to ThdF.** Fluorescence anisotropy of FAD (0.2 μM) at several concentrations of apoThdF. Fitting the binding curve with a cooperative binding model (Equation 3) resulted in a *K*_D_ = 6.8 ± 0.2 μM and n = 1.54 ± 0.05. Fitting with an uncooperative binding model (Hill coefficient set to 1) worsened the fit (*dashed red line*). Each data point represents the mean and SD of three technical replicates on one plate. The anisotropy measurement was performed independently three times on different days (data not shown). For *K*_D_ and n, the mean and SD of these three replicates are shown. FAD, flavin adenine dinucleotide.

To investigate the binding of FAD to the reductase, we generated apoThdF by removing the copurified FAD with 2 M KBr in a centrifugal concentrator. Since a large part (>50%) of the protein precipitated after removal of the FAD, the integrity of the remaining protein was checked and confirmed by analytical SEC. A binding curve for FAD was then determined by fluorescence anisotropy (Fig. 9).

Fitting the resulting curve with Equation 3 resulted in a dissociation constant of 6.8  ± 0.2 μM for the binding of FAD to ThdF (Fig. 9). This value matches the dissociation constant of a substrate flavin much better than that of a tightly bound cofactor in the case of a class I reductase catalyzing a ping-pong mechanism. For comparison, a dissociation constant of 11.7 μM was determined for the flavin reductase FerA (sequential mechanism) (8), while PheA2 (ping-pong mechanism) (27) with a *K*_D_ value of 10 nM has a much higher affinity for FAD. Other flavin reductases that also catalyze sequential mechanisms, such as PrnF (*K*_D_: 80 nM) (34), BorF (*K*_D_: 110 nM) (9), and AbeF (*K*_D_: 740 nM) (9), have higher affinities for FAD than ThdF, but these are still lower than the affinity of PheA2. In addition, for all these reductases catalyzing sequential mechanisms, the bound FAD can be removed with 2 M urea and 2 M KBr, which is not the case for PheA2 even with 4 M urea (27). Accordingly, the observed dissociation constant for the binding of FAD to ThdF further argues against a ping-pong mechanism and supports the sequential mechanism determined by steady-state kinetics.

Interestingly, the obtained Hill coefficient of n = 1.5 suggests a weak positive cooperative binding of FAD. This cooperative behavior could indicate an allosteric coupling between the two monomers in the dimer. Coupling between the two substrate binding sites in the dimer has already been observed for the reductase CobR (35), where this was associated with half-site reactivity. The authors discussed a solvent-containing channel at the dimer interface, connecting the two binding sites, as a possibility for a communication between them. In ThdF, there is also such a tunnel connecting the two flavin-binding sites (Fig. S11).

## Discussion

**Figure 10:**
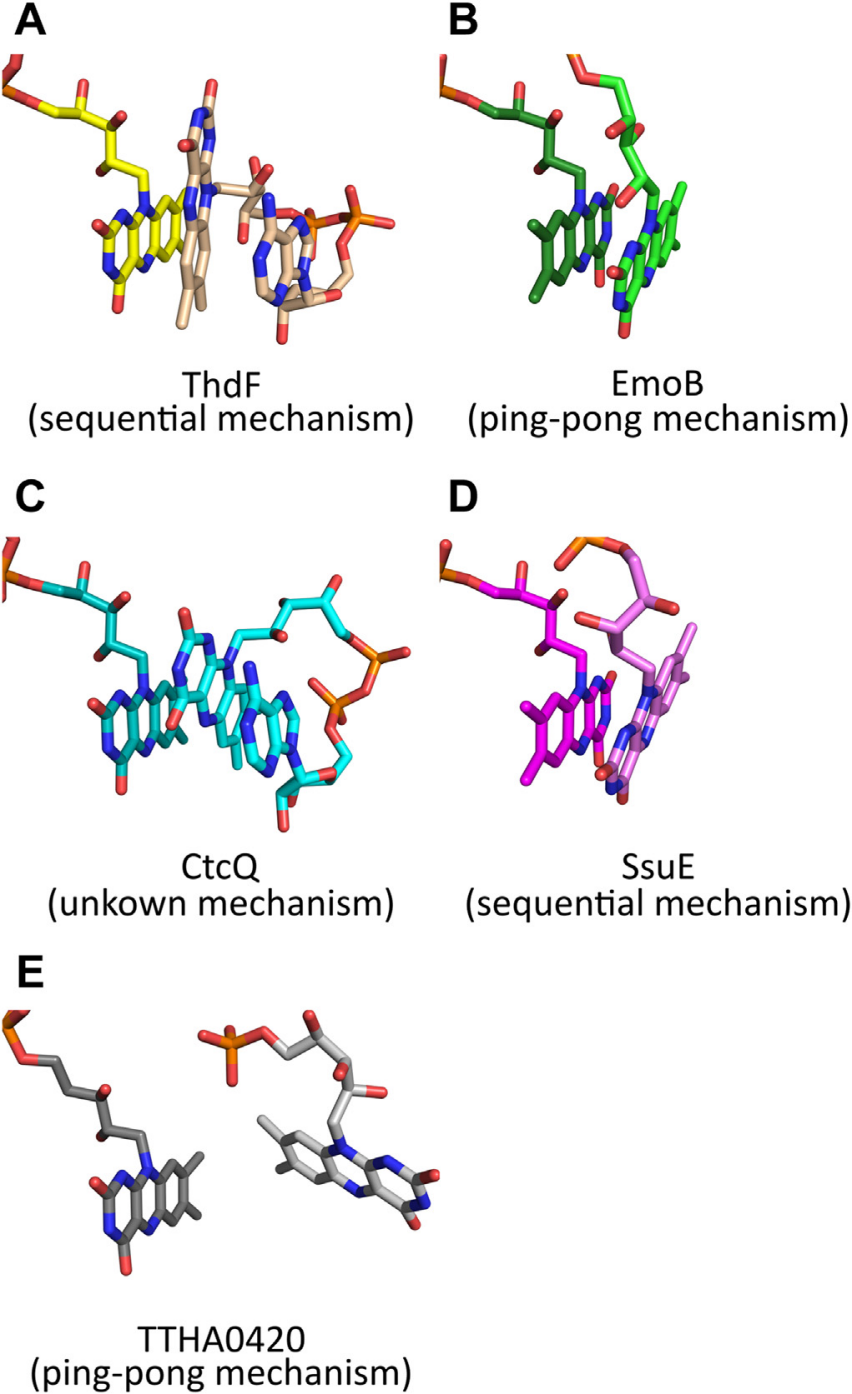
**Comparison of relative binding poses of the isoalloxazine moieties found in different flavin reductases.** Enzymes in the *left column* belong to the short-chain flavin reductase family (related to PheA2 and HpaC), enzymes in the *right column* belong to the flavodoxin-like family and reduce FMN. *A*, two FADs (*yellow and wheat carbon atoms*) bound in ThdF. *B*, prosthetic flavin (*dark green carbon atoms*) and more loosely bound FMN (*light green carbon atoms*) bound to the ping-pong reductase EmoB (PDB 4ltm). *C*, two FADs (*dark and light cyan carbon atoms*) found in CtcQ (PDB 8ct0, chain A). *D*, two bound FMNs (*dark and light magenta carbon atoms*) in SsuE (PDB 4ptz, chain A). *E*, bound cofactor FAD (*dark gray carbon atoms*) and substrate FAD (*light gray carbon atoms*), of which only the FMN moiety was modeled in TTHA0420 (PDB 1yoa). In all cases except for SsuE and TTHA0420 (no NAD-bound structures available), NADH binds instead of the *wheat or lighter colored flavins* according to the crystal structures (EmoB: PDB 4ltn, CtcQ: 8ct0, chains C and H). FAD, flavin adenine dinucleotide.

Two different crystal forms contain eight crystallographically independent ThdF molecules that all bind two FADs, suggesting a ping-pong mechanism. In contrast, our steady-state kinetics showed that flavin reduction in ThdF follows an ordered sequential mechanism, with FAD binding as the first substrate followed by NADH and the respective products being released in reverse order. To understand this apparent discrepancy, we searched for structures of other flavin reductases that bind two flavins. We found four such structures in the PDB, namely, EmoB (PDB 4ltm; (30)), SsuE (PDB 4ptz; (36)), CtcQ (PDB 8ct0), and TTHA0420 (PDB 1yoa; (31)). Of note, although EmoB and SsuE are also part of a two-component system, they belong to a different class of flavin reductases. While ThdF, CtcQ, and TTHA0420 are members of the short-chain flavin reductase family and can reduce FAD, EmoB and SsuE belong to the flavodoxin-like superfamily and are FMN reductases (36). In case of the ping-pong reductase EmoB, the double flavin-bound structure represents a functional state of the enzyme, since one flavin is permanently bound and acts as a prosthetic group (30). When comparing the relative orientation of the isoalloxazine rings, the flavin stacking in ThdF differs significantly. In ThdF, the two isoalloxazine rings are in parallel planes but orthogonal with ring III (two carbonyl moieties) of the stretched FAD (yellow carbon atoms) and ring I (two methyl groups) of the stacked FAD (wheat carbon atoms) being opposite of each other (Fig. 10*A*). This means that the central rings (ring II) are not directly above each other, which results in the two N5 atoms being relatively far apart (6.7 Å). In contrast, the isoalloxazine rings in EmoB (Fig. 10*B*) are almost perfectly antiparallel (rings I not facing each other) and the N5 atoms, which were assumed to mediate hydride transfer, lie directly on top of each other (distance of 3.4 Å) (30). The N5–N5 distance in ThdF is therefore roughly doubled compared to the distance during the proposed flavin–flavin hydride transfer found in EmoB as well as the C4–N5 distance (3.2–3.55 Å) normally found during a hydride transfer between nicotinamides and flavins (30, 37). These observations suggest that the orientation of the two isoalloxazine rings and the N5–N5 distance in ThdF are rather unsuitable for efficient hydride transfer. In CtcQ (Fig. 10*C*) and SsuE (Fig. 10*D*), the stacking of the flavins is comparable to EmoB, but the two ring systems are slightly twisted within the parallel plane. For SsuE, both isoalloxazine moieties are antiparallel to each other, and for CtcQ, they are parallel. The mechanism of CtcQ has not been published. Kinetic studies of SsuE suggest a sequential mechanism (36, 38), showing that flavins in general tend to bind to the NADH binding site, although this does not fit the reaction mechanism catalyzed by the protein. The situation is different in case of TTHA0420, which follows a ping-pong mechanism (Fig. 10*E*). Here, the planes of both isoalloxazine moieties are not parallel but they form a tilt angle of roughly 45°. This leads to a close proximity of both methylated rings I, which is thought to facilitate two single-electron transfers (31). Hence, the distance and orientation of the isoalloxazine moieties in ThdF are presumably unfavorable for efficient hydride transfer and argue against a ping-pong mechanism for flavin reduction in ThdF. We conclude that the double FAD-bound state of ThdF does most likely not reflect a productive state.

Interestingly, flavins are not the only cofactors that might bind in a nonproductive way to the NADH binding site. In a structure of the flavin reductase HpaC in complex with FAD and NADPH (PDB 2d38), the NADPH does not bind in the usual orientation required for electron transfer, but in the reverse orientation, with the adenine close to and the nicotinamide facing away from the flavin (28). An AlphaFold3 (39) prediction of the ThdF dimer in complex with FAD and NADPH leads to a similar reversed binding pose of NADPH (Fig. S12*A*). This presumably preferred but unproductive binding pose of NADPH could be the reason for the negligible activity of ThdF with NADPH (Fig. S12*B*). We assume that only a very small fraction of NADPH binds in an orientation that allows for hydride transfer from NADPH to the flavin. It is worth noting that AlphaFold3 predicts the binding of NAD^+^ and FAD to ThdF very similar to our structure (Fig. S13).

Taken together, these observations suggest that the binding site for the stacked (or nonprosthetic) cofactor of flavin reductases may not be highly specific for the binding of a particular cofactor in a particular orientation. Instead, at least under crystallographic conditions, other structurally similar cofactors may also bind, even if this may not be associated with a physiological function.

ThdF has a strikingly low *k*_cat_ value. One explanation for the low activity of ThdF could be the diluted concentration of ThdF used in the kinetic measurements. Investigation of ThdF with fluorescence anisotropy as a function of ThdF concentration revealed decreasing FAD fluorescence anisotropy with increasing dilution (Fig. S14). This indicated that FAD is leaving the protein at low concentrations. The departing cofactor could lead to destabilization of the protein, resulting in a lower amount of active enzyme in the assay. This assumption is also based on the observation that the addition of FAD during protein purification is necessary to achieve a high protein yield and that purified ThdF tends to aggregate once FAD is removed by KBr (2 M). Thermostability analysis of apoThdF and ThdF:FAD confirms this stabilizing effect of FAD at physiological salt concentrations (Fig. S10). A similar tendency is known for the corrin reductase CobR, which is not a flavin reductase but, as ThdF, can be classified into the HpaC-like group of flavin reductases regarding fold and sequence. For this enzyme, the absence of the FAD cofactor significantly reduced the thermostability (35).

Another possible explanation for the low activity of ThdF could be that the unproductive complex with a second FAD bound to the NADH binding site also forms in solution. It is possible that this low turnover in cooperation with Thal has the function of avoiding unproductive complexes that minimize halogenation efficiency. Indeed, it has been shown for Thal that binding of FADH_2_ in the absence of the substrate tryptophan results in the escape of the active halogenating agent hypohalous acid (15). Moreover, both RebH and Thal form unproductive complexes with FADH_2_ in the absence of O_2_ (15, 16). Thus, the slow turnover of FAD may be physiologically relevant for the Thal:ThdF system. Recently, for the flavin-dependent pyrrole halogenase PrnC, it was observed that a decrease in FAD concentration in halogenation assays led to a significant increase in substrate conversion, supporting the theory that a limited supply of reduced flavin can be beneficial for flavin-dependent halogenases (40).

The structural and kinetic data combined show that the extended FAD is the substrate FAD. This FAD interacts with ThdF mainly *via* its FMN moiety and the phosphate from AMP, while its adenosine moiety can adopt multiple conformations. Despite its flexibility, the adenosine moiety is not sufficiently exposed to allow simultaneous binding of Thal. Thus, an FAD-mediated complex between ThdF and the halogenase Thal appears unlikely if not impossible. Instead, our data suggest that if ThdF provides reduced flavin for Thal, it does so *via* free diffusion.

## Experimental procedures

### Protein expression

An *Escherichia coli* codon-optimized version of the *thdF* gene was synthesized by Invitrogen and cloned into a pETM-11 vector containing a TEV protease-cleavable His_6_-tag, using the NotI and NcoI restriction sites. The plasmid was transformed into *E. coli* BL21(DE3) cells by heat shock and an overnight culture was grown in LB medium (37 °C, 120 rpm) containing kanamycin (30 μg mL^−1^) as selection marker. The preculture was used to inoculate 2 L TB medium (30 μg mL^−1^ kanamycin) to an absorbance at 600 nm (*A*_600_) of 0.1. The main culture was incubated at 37 °C and 90 rpm until an *A*_600_ of 0.6, cooled down to 25 °C, and the expression of His_6_-TEV-ThdF was induced upon addition of 0.1 mM IPTG. The culture was incubated for further 20 h at 25 °C and 90 rpm before harvesting the cells *via* centrifugation (4500*g*, 45 min, 4 °C). The cell pellets were washed with cooled PBS and they were frozen at −20 °C until further use.

### Protein purification

A pellet from 2 L TB culture was resuspended in 50 ml cooled lysis buffer (50 mM Hepes pH 7.0, 250 mM NaCl, 15 mM imidazole pH 7.0, 100 μM FAD, and 1 mM 2-mercaptoethanol) and lysed in three cycles at 1.17 MPa using a SPCH ultrahigh-pressure cell homogenizer (Stansted Fluid Power). The soluble fraction was separated from insoluble compounds of the lysate by centrifugation (30,000*g*, 30 min, 4 °C). The soluble fraction was applied to a 1 ml His-Trap HP column (Cytiva) using an ÄKTA purifier system. The column was washed with 20 column volumes lysis buffer before eluting the protein in a gradient over 50 column volumes to 100% elution buffer (50 mM Hepes pH 7.0, 250 mM NaCl, 300 mM imidazole pH 7.0, 100 μM FAD, and 1 mM 2-mercaptoethanol). Elution fractions were pooled and dialyzed two times against 2 L dialysis buffer (50 mM Hepes pH 7.0, 250 mM NaCl, and 1 mM DTT) for 2 h and overnight at 20 °C. Before dialysis, TEV protease (1 mg TEV per 50 mg protein) was added to remove the His_6_-tag. The dialyzed protein was concentrated in a Vivaspin (Sartorius, 10 kDa molecular weight cut-off), and SEC was performed at 4 °C with SEC buffer (50 mM Hepes pH 7.0, 20 mM NaCl, and 1 mM DTT) using a 16/60 Superdex 200 column (GE Healthcare) again using an ÄKTA purifier system. Fractions containing TEV-digested ThdF were pooled, concentrated up to 20 mg mL^−1^ in a Vivaspin, and stored at −80 °C. In all purification steps, the protein had a bright yellow color.

### Crystallization

For crystallization, ThdF was diluted to 18 mg mL^−1^ with SEC buffer. All crystallization trials were set up at 20 °C in MRC 2-lens sitting-drop plates (SWISSCI) with 1:1 and 2:1 protein:reservoir drop ratios. Initially, crystallization of ThdF was screened using commercially available JCSG++ (Jena Bioscience) und MBclass Suite (Qiagen) screens. This resulted in bright yellow crystals in several conditions allowing structure determination up to a resolution of 2.9 Å. Aiming for a denser crystal packing, disordered regions of the protein were removed by *in situ* proteolysis. To this end, ThdF was mixed with chymotrypsin (CaCl_2_ added to a final concentration of 20 mM), trypsin, or elastase at a mass ratio of 1800:1 and incubated on ice for 10 min. Subsequently, crystallization plates were set up using JCSG++ (Jena Bioscience), Proplex Eco (Molecular Dimensions), and Morpheus (Molecular Dimension) screens. This yielded well-diffracting crystals in condition D12 of JCSG++ (40 mM KH_2_PO_4_, 20% (v/v) glycerol, and 16% (w/v) PEG 8000), resulting in crystal form 1 (space group *P*2_1_2_1_2_1_), and in condition B11 of Proplex Eco (0.1 M Hepes pH 7.0, 15% (w/v) PEG 4000), resulting in crystal form 2 (space group *P*2_1_).

Both conditions were used for cocrystallization and soaking attempts with different concentrations of NADH (up to 100 mM) to obtain a ThdF structure in complex with NADH. However, all measured crystals yielded structures with two bound FADs. The high-resolution structure of ThdF in complex with NADH and FAD in crystal form 1 (space group *P*2_1_2_1_2_1_) was obtained by soaking crystals (600 nl protein + 300 nl reservoir) in condition D12 of JCSG++ (40 mM KH_2_PO_4_, 20% (v/v) glycerol, 16% (w/v) PEG 8000) with 250 nl 100 mM NADH (dissolved in water) for 1 min and adding 250 nl 500 mM dithionite (dissolved in water) afterward. Immediately after adding dithionite, the crystals quickly and completely lost their yellow color and were harvested 1 to 5 min later. All crystals were flash-cooled in liquid nitrogen. Crystals from JCSG++ condition D12 were flash-cooled without additional cryoprotection, and crystals from Proplex Eco condition B11 were quickly soaked in reservoir solution containing 20% (v/v) PEG 400 before cooling.

### Structure determination

X-ray diffraction data of the FAD:FAD:ThdF complexes in both crystal forms were collected at 0.9763 Å on beamline P14 and the NADH:FAD:ThdF data at a wavelength of 0.9762 Å on beamline P13 both operated by EMBL Hamburg at the PETRA III storage ring (DESY) (41). Both beamlines were controlled using *MxCuBE* (42, 43), and all data were collected at 100 K. The diffraction data were processed using XDS (44) and STARANISO (45) to determine the diffraction limit with <*I/sd(I)* ≥ 1.20 as cut-off criterion. A structure from initial screening (2.90 Å, not deposited in the PDB) was phased *via* molecular replacement using *Phaser* (46), utilizing an ensemble of SgcE6 (PDB 4r82 and PDB 4hx6, sequence identity 31%) and BorF (PDB 5cho, sequence identity 29%). This early structure of ThdF was then used to phase the FAD:FAD:ThdF data in both crystal forms again by molecular replacement in *Phaser* each with four molecules per asymmetric unit forming two homodimers. The double FAD complex in crystal form 1 was solved in *P*2_1_2_1_2_1_ and in crystal form 2 in *P*2_1_. Both crystal forms have a different packing. The isomorphous (to crystal form 1) NADH-bound structure (NADH:FAD:ThdF) was solved by rigid body refinement with *phenix.refine* (47, 48) using a ligand-free version of the FAD:FAD:ThdF structure (crystal form 1). The models were optimized by several rounds of iterative model building in *Coot* (49) and restrained refinement using *phenix.refine*. The data and refinement statistics and information regarding anisotropic data cut-off are given in Tables S1–S3. PyMOL (www.pymol.org) was used to generate the structural illustrations.

### Steady-state kinetics

ThdF activity was assayed using steady-state kinetics by the initial velocity method. This was done spectroscopically at 25 °C in a plate reader (TECAN Spark 10M) by following the NADH turnover at 340 nm ε_340nm_ = 6.22 mM^−1^ cm^−1^. The reactions were started by addition of the enzyme at a final concentration of 50 nM. For the FAD-dependent measurements, the FAD concentration was varied from 0.125 to 8 μM at different NADH concentrations (25, 50, 75, 100 μM). For the NADH-dependent measurements, the NADH concentration was varied from 3.125 to 100 μM at different FAD concentrations (0.5, 0.75, 1, 2 μM). All values for initial velocity were determined in triplicate and corrected for the decreasing signal of the respective NADH concentration in the absence of the enzyme, which is caused by the spontaneous NADH oxidation under the aerobic conditions of the assay.

To investigate the detailed reaction mechanism including the order of substrate binding and product release, kinetic assays were performed with the addition of AMP, NMN, and NAD^+^ as inhibitors. Here, concentrations of one substrate were varied at a fixed concentration of the second substrate (1 μM FAD or 80 μM NADH) at different concentrations of the inhibitor (NMN: 0, 0.5, 1, 2 μM; AMP: 0, 1, 2, 4 μM; and NAD: 0, 40, 80, 120 μM).

### UV-visible spectroscopy

ThdF cofactor occupancy was studied with UV-visible spectroscopy. Absorption spectra of ThdF (12.5 μM) were recorded with a UV-visible spectrometer (UV-2450, Shimadzu) at room temperature using a Hellma absorption cuvette (10 mm path length). Spectra were obtained between 200 and 700 nm with a spectral resolution of 0.5 nm.

### Fluorescence spectroscopy

Fluorescence spectroscopy was used to study the fluorescence of ThdF-bound FAD. Sample concentrations were adjusted to an *OD*_450nm_ = 0.05 *via* UV-visible spectroscopy. Spectra were measured in a Jasco FP-8300 fluorescence spectrometer at 20 °C using a fluorescence cuvette (Hellma QS, 10 × 2 mm). For fluorescence emission spectra, excitation was at 450 nm with a bandwidth of 5 nm and the emission was detected from 470 to 700 nm with a scan speed of 1000 nm/min and a bandwidth of 5 nm at medium detector sensitivity.

### Fluorescence anisotropy

Fluorescence anisotropy (A) was used to study the binding of FAD to apoThdF at a fixed flavin concentration (0.2 μM) in the presence of different protein concentrations (0.1–190 μM). Experiments were performed in triplicate in a black 384-well plate (Brand, polystyrene, flat bottom) at 25 °C. Fluorescence anisotropy signals were measured in a plate reader (TECAN Spark 10M) containing a polarization filter for excitation at 430 nm, and the emission was detected at 530 nm. The polarization (P) was calculated from the fluorescence intensity of the emitted light parallel to the excitation (I_∥_) and the fluorescence intensity of the emitted light perpendicular to the excitation (I_⊥_) using Equation 1. G is a correction factor that corrects for instrument bias and is calculated by using the sample buffer as a reference blank.

(1) $$P=\left(I_{\|}-G\cdot I_{┴}\right)/\left(I_{\|}+G\cdot I_{┴}\right)$$

Values for fluorescence anisotropy (A) were calculated using Equation 2.

(2) A=2P/(3−P)

To determine the dissociation constant K_D_ for the flavin, the measured fluorescence anisotropy values A_O_ as a function of the protein concentration [apoThdF] were fitted using Equation 3. A_F_ and A_B_ are the fluorescence anisotropy of the free and protein-bound flavin, respectively, Q is the quenching factor, and n is the Hill coefficient.

(3) $$A_{O}=\frac{A_{F}+\left(A_{B}\cdot Q-A_{F}\right)\cdot \frac{{\left[\text{apoThdF}\right]}^{n}}{K_{D}^{n}+{\left[\text{apoThdF}\right]}^{n}}}{1+\left(Q-1\right)\cdot \frac{{\left[\text{apoThdF}\right]}^{n}}{K_{D}^{n}+{\left[\text{apoThdF}\right]}^{n}}}$$

This expression results from Equation 4 (50), which describes how the concentration of the protein-bound flavin (F_B_) is calculated from $A_{O}$, when equated with the Hill equation and converted to A_O_.

(4) $$F_{B}=\frac{A_{O}-A_{F}}{{\left(A_{B}-A_{O}\right)Q+A}_{O}-A_{F}}$$

### Redox titration of ThdF with dithionite

For reduction, 125 μM FAD bound to ThdF in 50 mM sodium phosphate buffer, pH 7.4, with 100 mM NaCl were titrated with a solution of 1.5 mM sodium dithionite in the same buffer. The dithionite solution was prepared to be anaerobic by three cycles of freeze–pump–thaw using the Schlenk technique with Argon as inert gas. The protein sample was prepared to be anaerobic by Argon influx for 2 h in a 1 cm × 1 cm cuvette with septum equipped with a magnetic stirrer. The dithionite solution was added in 20 μl steps, and UV-visible spectra were measured using a HR2000+ spectrometer with a DH-2000-BAL light source (Ocean Optics).

### Differential scanning fluorimetry

The melting temperatures of apoThdF and ThdF:FAD (final concentration 0.2 mg/ml) in 50 mM Hepes pH 8.0 at different NaCl concentrations (20–1000 mM) were determined by differential scanning fluorimetry using SyproOrange (final concentration: 5x) as a fluorescent dye. Melting curves were recorded in a real-time PCR thermocycler (Applied Biosystems StepOnePlus) in a linear gradient of 25 to 95 °C with continuous heating of 0.5 °C/min. Data analysis for melting point determination was performed by fitting the melting curves using a Boltzmann fit. All melting points were determined from measurements of three wells per sample on the same plate.

### Nano-ESI quadrupole TOF-MS

Nano-ESI measurements were performed using a quadrupole ion mobility spectrometry/TOF mass spectrometer (Synapt G2Si; Waters GmbH) in resolution mode, interfaced to a nano-ESI ion source. Nitrogen served both as the nebulizer gas and the dry gas for nano-ESI. Nitrogen was generated by a nitrogen generator NGM 11. Argon serves as collision gas for *top-down* MS/MS experiments. For *top-down* MS/MS experiments, ions of interest were isolated with the quadrupole and fragmented by *collision-induced dissociation* in the transfer cell of the mass spectrometer. Fragment ions were detected using the ToF analyzer of the instrument. Purified ThdF was buffer exchanged against 50 mM ammonium acetate and used for nano-ESI-Q-ToF-MS measurements under native conditions (final protein concentration: 10 μM). Under denaturing conditions, ThdF was dissolved in 50% acetonitrile supplemented with 0.1% formic acid (final protein concentration: 10 μM). The sample was introduced by static nano-ESI using in-house pulled glass emitters. The mass axis was externally calibrated with ESI-L Low Concentration Tuning Mix (Agilent Technologies) as calibration standard.

Scan accumulation and data processing was performed with MassLynx 4.1 (Waters GmbH) on a PC workstation. The spectra shown here were generated by the accumulation and averaging of several single spectra. Determination of protein masses was performed using raw data when using the tool MaxEnt1 (Waters GmbH) or baseline subtracted, smoothed, and centroided data when using the tool Transform (Waters GmbH). Fragment mass spectra were processed using the tool MaxEnt3 (Waters GmbH), and MS/MS fragment analysis was performed using the software BioLynx (Waters GmbH).

## Data availability

Coordinates and structure factors were deposited in the PDB with accession codes 9fd4 (FAD:FAD:ThdF in crystal form 1), 9fd5 (FAD:FAD:ThdF in crystal form 2), and 9fd6 (NADH:FAD:ThdF). Diffraction images are available from the SBGrid Data Bank (51) with accession numbers 1109 (FAD:FAD:ThdF in crystal form 1), 1110 (FAD:FAD:ThdF in crystal form 2), and 1111 (NADH:FAD:ThdF).

## Supporting information

This article contains supporting information (33, 45, 52, 53, 54).

## Conflict of interest

The authors declare that they have no conflicts of interest with the contents of this article.
