# Supplementary material for: The NADH-dependent flavin reductase ThdF follows an ordered sequential mechanism though crystal structures reveal two FAD molecules in the active site
Source: J Biol Chem. 2024 Dec 24;301(2):108128. doi: 10.1016/j.jbc.2024.108128 (PMC11795597; doi:10.1016/j.jbc.2024.108128)
Supplement: Supporting information [file mmc1.docx]

Supplementary Information

The NADH-dependent flavin reductase ThdF follows an ordered sequential mechanism though crystal structures reveal two FAD molecules in the active site

Hendrik J. Horstmeier,^1✝^ Simon Bork,^1✝^ Marius F. Nagel,^1^ Willy Keller,^1^ Jens Sproß,^2^ Niklas Diepold,^3,4^ Marie Ruppel,^1^ Tilman Kottke,^3,4^ and Hartmut H. Niemann^1^

^1^ Structural Biochemistry, Department of Chemistry, Bielefeld University, Universitätsstraße 25, 33615 Bielefeld, Germany

^2^ Industrial Organic Chemistry and Biotechnology – Mass Spectrometry, Department of Chemistry, Bielefeld University, Universitätsstraße 25, 33615 Bielefeld, Germany

^3^Biophysical Chemistry and Diagnostics, Department of Chemistry, Bielefeld University, Universitätsstraße 25, 33615 Bielefeld, Germany

^4^Biophysical Chemistry and Diagnostics, Medical School OWL, Bielefeld University, Universitätsstraße 25, 33615 Bielefeld, Germany

^✝^ These authors contributed equally to this work.

* For correspondence: Hartmut Niemann: hartmut.niemann@uni-bielefeld.de

# Content

- Gene sequences
- Equation S1
- Tables S1 to S4
- Figures S1 to S14

**Gene sequences**

*thdF*

ATGAAACATCACCATCACCATCACCCCATGAGCGATTACGACATCCCCACTACTGAGAATCTTTATTTTCAGGGCGCCATGGCTGGTGCAGCCGGTGGTGTTACCACCGAACAGCAGCATTATCGTGATCTGATGAGCGCATTTCCGACCGGTATTGCAGTTGTTACCAGCCTGGATGCACAGGGTGTTCCGCGTGGTATGACCTGTAGCAGCGTTACCAGCGCAACCCTGAGTCCGCCTACACTGCTGGTTTGTCTGCGTAATGGTAGCGCGACCCTGGATGCCGTTAGTGCAACCCGTGGTTTTGCAGTTAATCTGCTGCATGATGGTGGTCGTCATGCAGCCGAAGTTTTTAGCGGTCCGGATCCGAATCGTTTTAGCCGTGTTCAGTGGAAACAGTGTCGTAGCGGTCTGCCGTGGCTGAGCAAAGATGCATTTGCAGTTGCAGAATGTCGTGTTAGCGGCACCCAAGAAGTTGGCGATCATACCGTTGTTTTTGGTGAAGTTGCACGTATTGCACAGACCGATGGTACACCGCTGCTGTATGGTCTGCGTAGCTTTGCAGCATGGCCTCTGCCGAGCGAAAGCGCACGTCAGGGTAGCGAAGCAGCACCGCGTGGTTCAGAACCGGCATATGGTGCACGTAGCAGCCGTGGTTAA

*thdF_1-168_*

ATGAAACATCACCATCACCATCACCCCATGAGCGATTACGACATCCCCACTACTGAGAATCTTTATTTTCAGGGCGCCATGGCTGGTGCAGCCGGTGGTGTTACCACCGAACAGCAGCATTATCGTGATCTGATGAGCGCATTTCCGACCGGTATTGCAGTTGTTACCAGCCTGGATGCACAGGGTGTTCCGCGTGGTATGACCTGTAGCAGCGTTACCAGCGCAACCCTGAGTCCGCCTACACTGCTGGTTTGTCTGCGTAATGGTAGCGCGACCCTGGATGCCGTTAGTGCAACCCGTGGTTTTGCAGTTAATCTGCTGCATGATGGTGGTCGTCATGCAGCCGAAGTTTTTAGCGGTCCGGATCCGAATCGTTTTAGCCGTGTTCAGTGGAAACAGTGTCGTAGCGGTCTGCCGTGGCTGAGCAAAGATGCATTTGCAGTTGCAGAATGTCGTGTTAGCGGCACCCAAGAAGTTGGCGATCATACCGTTGTTTTTGGTGAAGTTGCACGTATTGCACAGACCGATGGTACACCGCTGCTGTATGGTCTGCGTAGCTTTGCAGCATGGCCTCTGCCGAGCTAA

**Steady-state kinetics for ordered sequential mechanism**

$v=v_{max}\left( \frac{\left[ A \right]\left[ B \right]}{K_{A}K_{B}}+\frac{\left[ B \right]}{K_{B}}+1 \right)$ (Equation S1)

Equation S1 (33) gives the steady-state rate equation for an ordered sequential mechanism that we propose for the flavin reductase ThdF. *v* and *v*_max_ are the initial and maximum rates, respectively. A is the first binding substrate and B is the second binding substrate, where *K*_A_ and *K*_B_ are the corresponding dissociation constants.

**Table S1: Data collection and processing.** Values for the outer shell are given in parentheses.

| Structure | FAD:FAD:ThdF  crystal form 1 | FAD:FAD:ThdF  crystal form 2 | NADH:FAD:ThdF |
| --- | --- | --- | --- |
| PDB | 9fd4 | 9fd5 | 9fd6 |
| DOI of raw data (images) | 10.15785/SBGRID/1109 | 10.15785/SBGRID/1110 | 10.15785/SBGRID/1111 |
| Diffraction source | DESY P14 | DESY P14 | DESY P13 |
| Wavelength (Å) | 0.9763 | 0.9763 | 0.9762 |
| Temperature (K) | 100 | 100 | 100 |
| Detector | EIGER2 CdTe 16M | EIGER2 CdTe 16M | EIGER X16M |
| Crystal-detector distance (mm) | 160.28 | 180.011 | 178.674 |
| Rotation range per image (°) | 0.10 | 0.15 | 0.10 |
| Total rotation range (°) | 360 | 360 | 360 |
| Exposure time per image (s) | 0.008 | 0.008 | 0.008 |
| Space group | *P*2_1_2_1_2_1_ | *P*2_1_ | *P*2_1_2_1_2_1_ |
| *a*, *b*, *c* (Å) | 47.79, 83.61, 157.34 | 59.15, 77.37, 74.49 | 47.76, 83.48, 159.06 |
| α, β, γ (°) | 90.0, 90.0, 90.0 | 90.0, 111.8, 90.0 | 90.0, 90.0, 90.0 |
| Mosaicity (°) | 0.057 | 0.063 | 0.089 |
| Resolution range (Å) | 57.30 – 1.14  (1.22 – 1.14) | 70.07 – 1.31  (1.37 – 1.31) | 73.92 – 1.43  (1.55 – 1.43) |
| Total No. of reflections | 6651216 (203781) | 895455 (37055) | 1297557 (66054) |
| No. of unique reflections | 188862 (9443) | 129944 (6497) | 95935 (4797) |
| Completeness spherical (%) | 82.4 (22.5) | 86.9 (35.6) | 80.3 (18.6) |
| Completeness ellipsoidal (%) | 93.8 (52.4) | 93.6 (59.7) | 95.5 (60.2) |
| Redundancy | 35.2 (21.6) | 6.9 (5.7) | 13.5 (13.8) |
| 〈 *I*/σ(*I*)〉 | 21.5 (1.9) | 16.3 (2.5) | 14.9 (1.5) |
| CC_1/2_ | 0.999 (0.624) | 0.999 (0.804) | 0.999 (0.594) |
| Rmeas (%) | 0.089 (2.242) | 0.062 (0.706) | 0.098 (1.974) |
| Overall *B* from Wilson plot (Å^2^) | 16.04 | 11.03 | 18.12 |

**Table S2: Structure solution and refinement.** Values for the outer shell are given in parentheses.

| Name Dataset | FAD:FAD:ThdF  crystal form 1 | FAD:FAD:ThdF  crystal form 2 | NADH:FAD:ThdF |
| --- | --- | --- | --- |
| PDB | 9fd4 | 9fd5 | 9fd6 |
| Resolution range (Å) | 57.29 – 1.14  (1.15 – 1.14) | 54.07 – 1.31  (1.33 – 1.31) | 45.74 – 1.43  (1.44 – 1.43) |
| Completeness (%) | 82.37 (3.75) | 86.86 (11.91) | 80.27 (0.43) |
| No. of reflections, working set | 188799 (281) | 129927 (589) | 95908 (17) |
| No. of reflections, test set | 9435 (21) | 6445 (37) | 4919 (2) |
| Final *R*_work_ (%) | 12.09 (24.49) | 11.52 (21.76) | 14.58 (31.78) |
| Final *R*_free_ (%) | 14.26 (31.69) | 15.28 (25.95) | 18.22 (26.14) |
| No. of non-H atoms | 6319 | 6515 | 6374 |
| Protein | 5174 | 5342 | 5125 |
| Ligand | 568 | 477 | 573 |
| Water | 577 | 696 | 676 |
| R.m.s. deviations |  |  |  |
| Bonds (Å) | 0.007 | 0.008 | 0.004 |
| Angles (°) | 1.11 | 1.06 | 0.85 |
| Average *B* factors (Å^2^) | 22.74 | 17.02 | 22.39 |
| Protein | 21.31 | 15.11 | 20.92 |
| Ligand | 21.97 | 15.80 | 21.96 |
| Water | 36.36 | 32.49 | 33.91 |
| Ramachandran plot |  |  |  |
| Most favoured (%) | 98.74 | 98.89 | 97.95 |
| Allowed (%) | 1.26 | 1.11 | 2.05 |
| Outliers (%) | 0 | 0 | 0 |

**Table S3. Metrics regarding anisotropy.** Anisotropy ratio, anisotropic S/N ratio (explanation below) and worst and best diffraction limit after cut-off given by the *STARANISO* server for each dataset. For comparison of anisotropic (*STARANISO*) and isotropic (*XSCALE*) scaling and truncation 〈*I/σ(I)*〉 and CC_1/2_ are summarized (values for the outer shell are given in parentheses). The resolution during isotropic scaling was limited in *XSCALE* to the highest resolution given by *STARANISO*.

| **Structure** | | **9fd4** | **9fd5** | **9fd6** |
| --- | --- | --- | --- | --- |
| **Anisotropy ratio** | | 0.110 | 0.743 | 0.275 |
| **Anisotropic S/N ratio** | | 5.07 | 16.61 | 5.75 |
| **Worst diffraction limit after cut-off / Å** | | 1.350 | 1.560 | 1.646 |
| **Best diffraction limit after cut-off / Å** | | 1.141 | 1.315 | 1.425 |
| **anisotropic scaling (*STARANISO*)** | **〈*I/σ(I)*〉** | 21.5 (1.9) | 16.3 (2.5) | 14.9 (1.5) |
|  | **CC_1/2_** | 0.999 (0.624) | 0.999 (0.804) | 0.999 (0.594) |
| **isotropic scaling (*xscale*)** | **〈*I/σ(I)*〉** | 18.79 (0.75) | 15.44 (1.65) | 12.21 (0.50) |
|  | **CC_1/2_** | 0.999 (0.252) | 0.999 (0.659) | 0.999 (0.204) |

**Definitions from STARANISOs *GLOSSARY of terminology related to anisotropy* (45):**

**Anisotropy ratio:** Difference between the largest and smallest eigenvalues of the overall anisotropy U tensor divided by *U*_eq_: (*E*_max_ - *E*_min_) / *U*_eq_. This metric lies between zero (isotropic) and 3 (maximally anisotropic).

**Anisotropic S/N ratio:** The maximum value over all observed reflections of the absolute deviation of the squared anisotropy correction factor from 1, multiplied by the local mean intensity/standard uncertainty ratio: max*_h_*(|exp(4π^2^*s_h_*^T^ΔU*s_h_*)-1|<*I_h_*/σ(*I****_h_***)>). Unlike the anisotropy ratio and the fraction anisotropy this takes into account both the fact that large anisotropies represent larger differences in intensity at high *d** and that there will be a greater contribution from reflections with a high value of the local mean*I*/σ(*I*). This metric is zero in the isotropic case, with no limit in the anisotropic case.

**
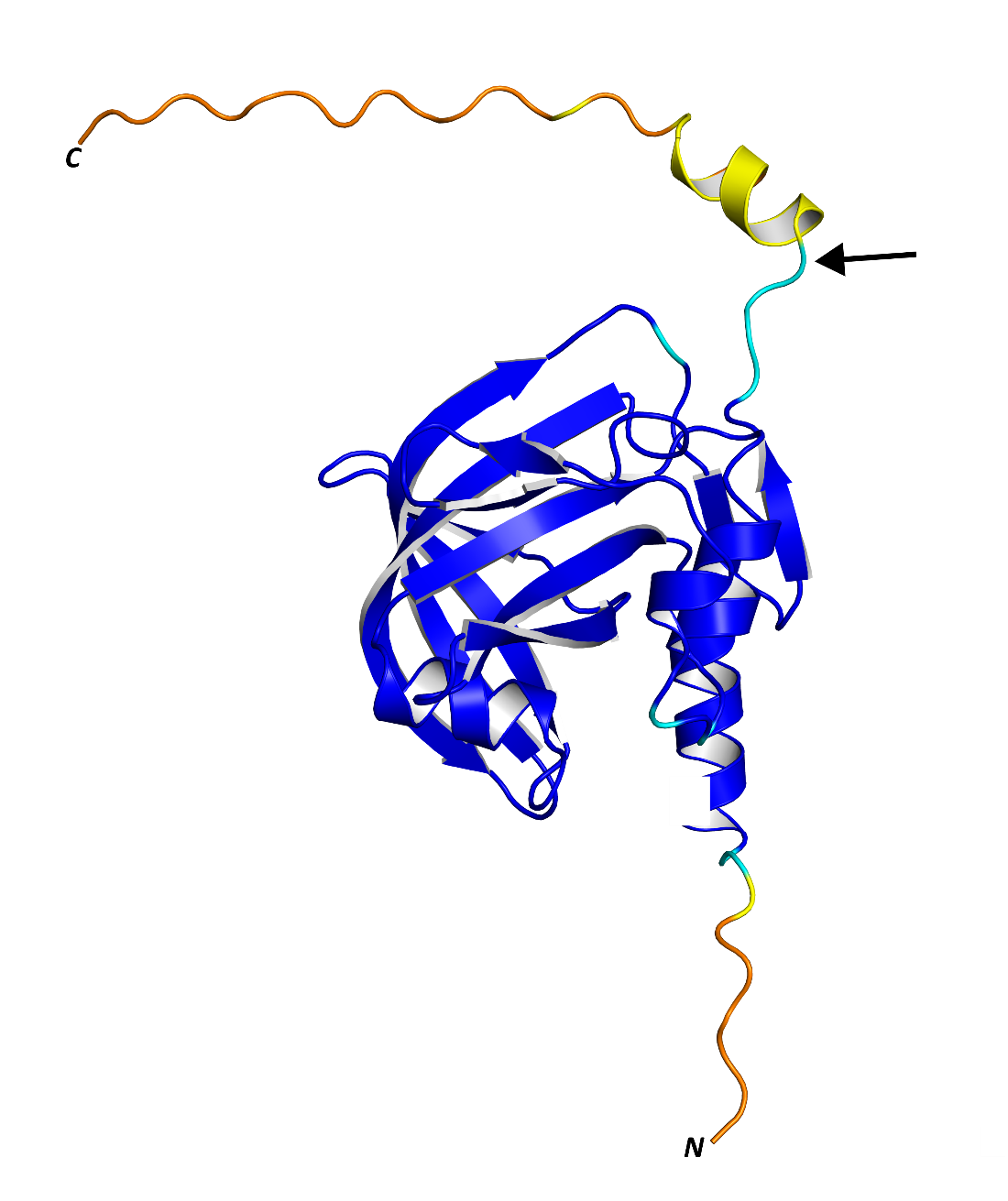
**

**Figure S1: AlphaFold2 prediction for ThdF.** Model AF-A0A1B1V585-F1 with labeled N- and C-terminus and an arrow indicating amino acid 168, which is the last one visible in all crystal structures.

**
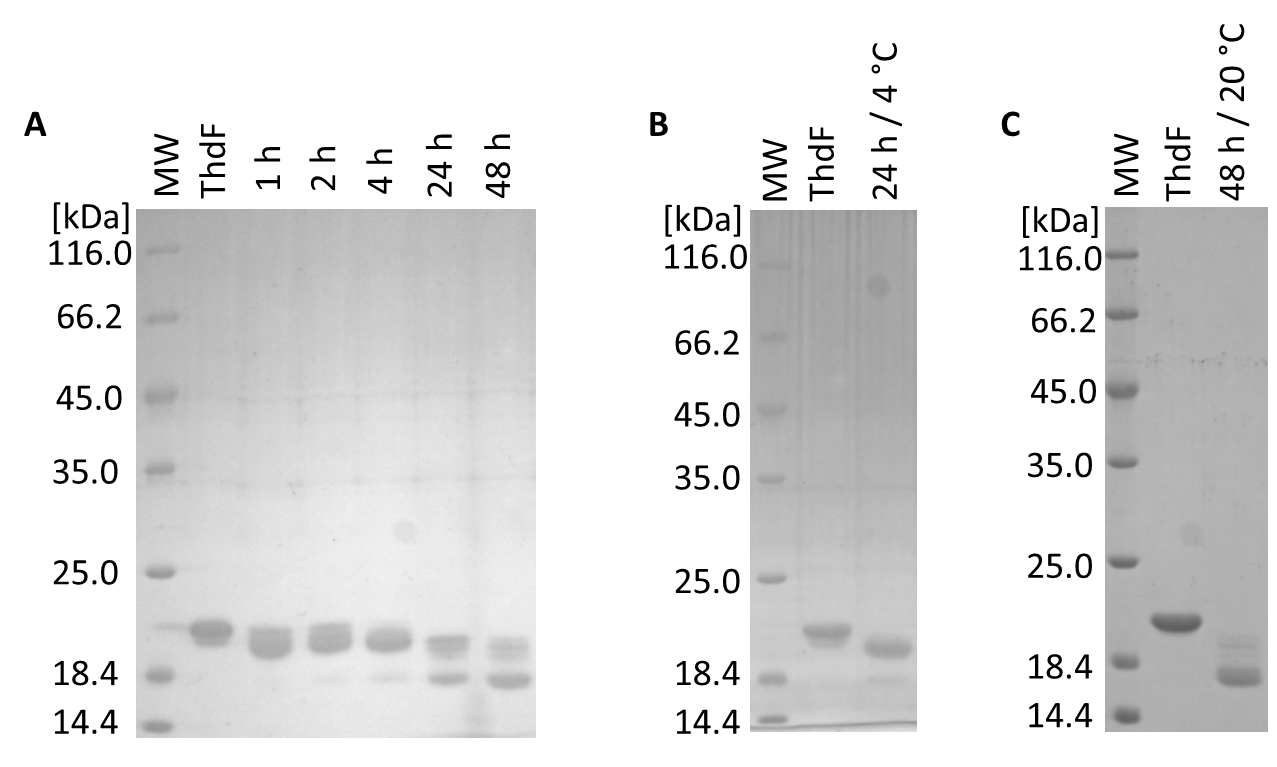
**

**Figure S2: ThdF digestion with elastase.** (**A**) Kinetics of ThdF digestion with elastase. ThdF was digested with elastase under the same conditions as for crystallization (20 °C and ThdF:elastase ratio). SDS-PAGE analysis showed different cleavage products over time. In the first two hours, mainly slightly smaller cleavage products occur, while at later time points (24 h and 48 h) cleavage products around 18 kDa (according to the gel) are most prominent. (**B**) Sample prepared of the heavier / earlier cleavage product analyzed by mass spectrometry. (**C**) Sample prepared of the lighter / later cleavage product analyzed by mass spectrometry. SDS-PAGE was performed with 15 % polyacrylamide gels. Staining was performed using a Coomassie Brilliant Blue R250 (0.25 %) solution.


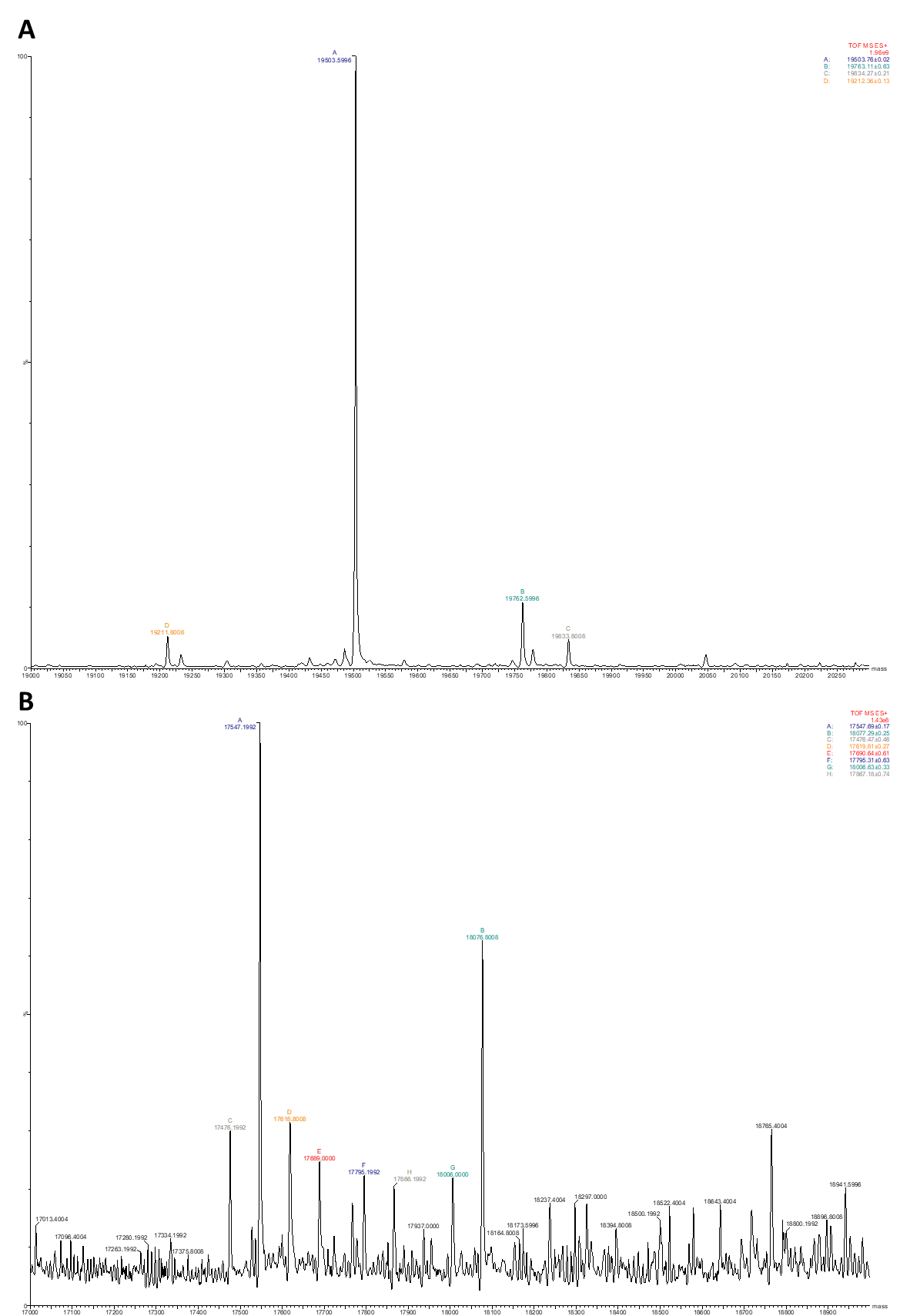


**Figure S3: Results of MS analysis of elastase digested ThdF.** (**A**) ThdF digested with elastase for 24 h at 4 °C (Fig. S2 B). (**B**) ThdF digested with elastase for 48 h at 20 °C (Fig. S2 C). Both panels show deconvoluted results of the MS analysis under denaturing conditions after processing with MaxEnt1.

**Table S4: Identified cleavage products of ThdF using mass spectrometry under denaturing conditions (Fig. S3).**

| Sequence of full-length ThdF after TEV cleavage of the His_6_-tag. The first two residues GA (shown in italics) do not belong to the native ThdF sequence but are due to the restriction sites used for cloning. The first methionine is residue 1 of native ThdF. The longest sequence resolved in any of our crystal structures is highlighted in green. It spans residues Gly6 to Ser168 of native ThdF.  Theoretical mass: 20306.72 Da | | |
| --- | --- | --- |
| *GA*MAGAAGGVTTEQQHYRDLMSAFPTGIAVVTSLDAQGVPRGMTCSSVTSATLSPPTLLVCLRNGSATLDAVSATRGFAVNLLHDGGRHAAEVFSGPDPNRFSRVQWKQCRSGLPWLSKDAFAVAECRVSGTQEVGDHTVVFGEVARIAQTDGTPLLYGLRSFAAWPLPSESARQGSEAAPRGSEPAYGARSSRG | | |
| NanoESI-Q-ToF-MS analysis enabled the identification of amino acid sequences of various ThdF fragments obtained after elastase treatment (highlighted in yellow). | | |
| **ThdF after elastase cleavage for 24 hr at 4 °C (Fig. S2 B)** | | |
| **Series** | **m(exp)** | **m(theo)** |
| Series A (**main species**) | 19503.76 +/- 0.02 Da | 19503.86 Da |
| GAMAGAAGGVTTEQQHYRDLMSAFPTGIAVVTSLDAQGVPRGMTCSSVTSATLSPPTLLVCLRNGSATLDAVSATRGFAVNLLHDGGRHAAEVFSGPDPNRFSRVQWKQCRSGLPWLSKDAFAVAECRVSGTQEVGDHTVVFGEVARIAQTDGTPLLYGLRSFAAWPLPSESARQGSEAAPRGSEPAYGARSSRG | | |
| Series B | 19763.11 +/- 0.63 Da | 19763.31 Da |
| GAMAGAAGGVTTEQQHYRDLMSAFPTGIAVVTSLDAQGVPRGMTCSSVTSATLSPPTLLVCLRNGSATLDAVSATRGFAVNLLHDGGRHAAEVFSGPDPNRFSRVQWKQCRSGLPWLSKDAFAVAECRVSGTQEVGDHTVVFGEVARIAQTDGTPLLYGLRSFAAWPLPSESARQGSEAAPRGSEPAYGARSSRG | | |
| Series C | 19834.27 +/- 0.21 Da | 19834.33 Da |
| GAMAGAAGGVTTEQQHYRDLMSAFPTGIAVVTSLDAQGVPRGMTCSSVTSATLSPPTLLVCLRNGSATLDAVSATRGFAVNLLHDGGRHAAEVFSGPDPNRFSRVQWKQCRSGLPWLSKDAFAVAECRVSGTQEVGDHTVVFGEVARIAQTDGTPLLYGLRSFAAWPLPSESARQGSEAAPRGSEPAYGARSSRG | | |
| Series D | 19212.36 +/- 0.13 Da | 19212.67 Da |
| GAMAGAAGGVTTEQQHYRDLMSAFPTGIAVVTSLDAQGVPRGMTCSSVTSATLSPPTLLVCLRNGSATLDAVSATRGFAVNLLHDGGRHAAEVFSGPDPNRFSRVQWKQCRSGLPWLSKDAFAVAECRVSGTQEVGDHTVVFGEVARIAQTDGTPLLYGLRSFAAWPLPSESARQGSEAAPRGSEPAYGARSSRG | | |

| **ThdF after elastase cleavage for 48 hr at 20 °C (Fig. S2 C)** | | |
| --- | --- | --- |
| **Series** | **m(exp)** | **m(theo)** |
| Series A (**main species**) | 17547.69 +/- 0.17 Da | 17547.76 Da |
| GAMAGAAGGVTTEQQHYRDLMSAFPTGIAVVTSLDAQGVPRGMTCSSVTSATLSPPTLLVCLRNGSATLDAVSATRGFAVNLLHDGGRHAAEVFSGPDPNRFSRVQWKQCRSGLPWLSKDAFAVAECRVSGTQEVGDHTVVFGEVARIAQTDGTPLLYGLRSFAAWPLPSESARQGSEAAPRGSEPAYGARSSRG | | |
| Series B | 18077.29 +/- 0.25 Da | 18077.37 Da |
| GAMAGAAGGVTTEQQHYRDLMSAFPTGIAVVTSLDAQGVPRGMTCSSVTSATLSPPTLLVCLRNGSATLDAVSATRGFAVNLLHDGGRHAAEVFSGPDPNRFSRVQWKQCRSGLPWLSKDAFAVAECRVSGTQEVGDHTVVFGEVARIAQTDGTPLLYGLRSFAAWPLPSESARQGSEAAPRGSEPAYGARSSRG | | |
| Series C | 17476.47 +/- 0.46 Da | 17476.68 Da |
| GAMAGAAGGVTTEQQHYRDLMSAFPTGIAVVTSLDAQGVPRGMTCSSVTSATLSPPTLLVCLRNGSATLDAVSATRGFAVNLLHDGGRHAAEVFSGPDPNRFSRVQWKQCRSGLPWLSKDAFAVAECRVSGTQEVGDHTVVFGEVARIAQTDGTPLLYGLRSFAAWPLPSESARQGSEAAPRGSEPAYGARSSRG | | |
| Series D | 17619.81 +/- 0.27 Da | 17618.84 Da |
| GAMAGAAGGVTTEQQHYRDLMSAFPTGIAVVTSLDAQGVPRGMTCSSVTSATLSPPTLLVCLRNGSATLDAVSATRGFAVNLLHDGGRHAAEVFSGPDPNRFSRVQWKQCRSGLPWLSKDAFAVAECRVSGTQEVGDHTVVFGEVARIAQTDGTPLLYGLRSFAAWPLPSESARQGSEAAPRGSEPAYGARSSRG  Or:  GAMAGAAGGVTTEQQHYRDLMSAFPTGIAVVTSLDAQGVPRGMTCSSVTSATLSPPTLLVCLRNGSATLDAVSATRGFAVNLLHDGGRHAAEVFSGPDPNRFSRVQWKQCRSGLPWLSKDAFAVAECRVSGTQEVGDHTVVFGEVARIAQTDGTPLLYGLRSFAAWPLPSESARQGSEAAPRGSEPAYGARSSRG | | |
| Series E | 17690.64 +/- 0.61 Da | 17689.91 Da |
| GAMAGAAGGVTTEQQHYRDLMSAFPTGIAVVTSLDAQGVPRGMTCSSVTSATLSPPTLLVCLRNGSATLDAVSATRGFAVNLLHDGGRHAAEVFSGPDPNRFSRVQWKQCRSGLPWLSKDAFAVAECRVSGTQEVGDHTVVFGEVARIAQTDGTPLLYGLRSFAAWPLPSESARQGSEAAPRGSEPAYGARSSRG | | |


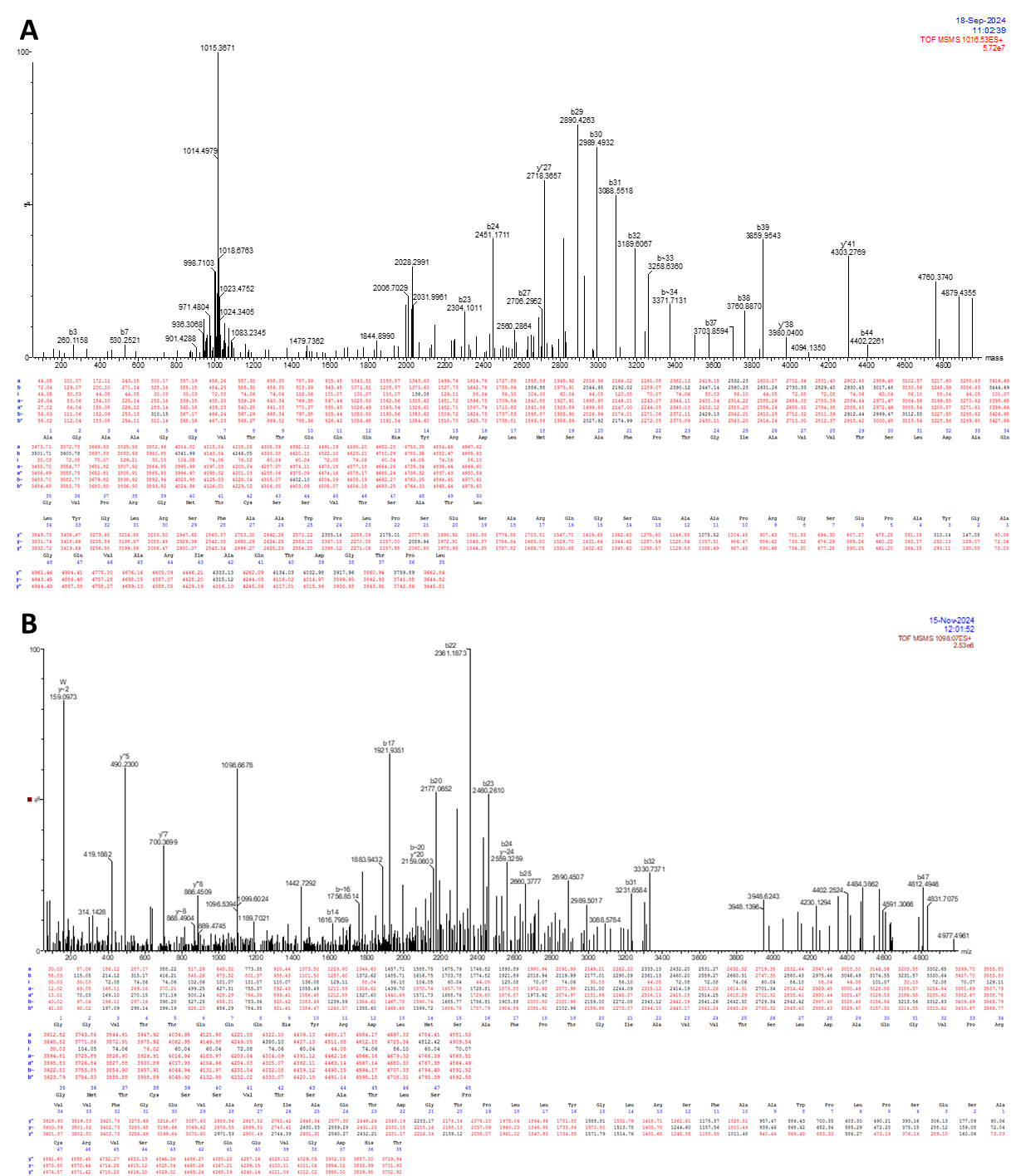
**Figure S4: Results of *top-down* MS/MS analysis of the two main species of both analyzed elastase digested ThdF samples.** (**A**) Analysis of the 18-fold charged ion (m/z 1084.6) from series A of the earlier/heavier cleavage product (see Fig. S3 A). (**B**) Analysis of the 16-fold charged ion (m/z 1097.7) from series A of the later/lighter cleavage product (see Fig. S3 B). Both panels show fragment mass spectra processed using MaxEnt3 and MS/MS fragment analysis using BioLynx. Fragment ions matched by BioLynx to the amino acid sequences are highlighted in black. *N-terminal* b-ions *and C-terminal* y-ions confirm the sequence of the *N-* and *C-termini*. Fragment ions indexed with “~”: additional water loss, fragment ions indexed with “*”: additional ammonia loss.

**
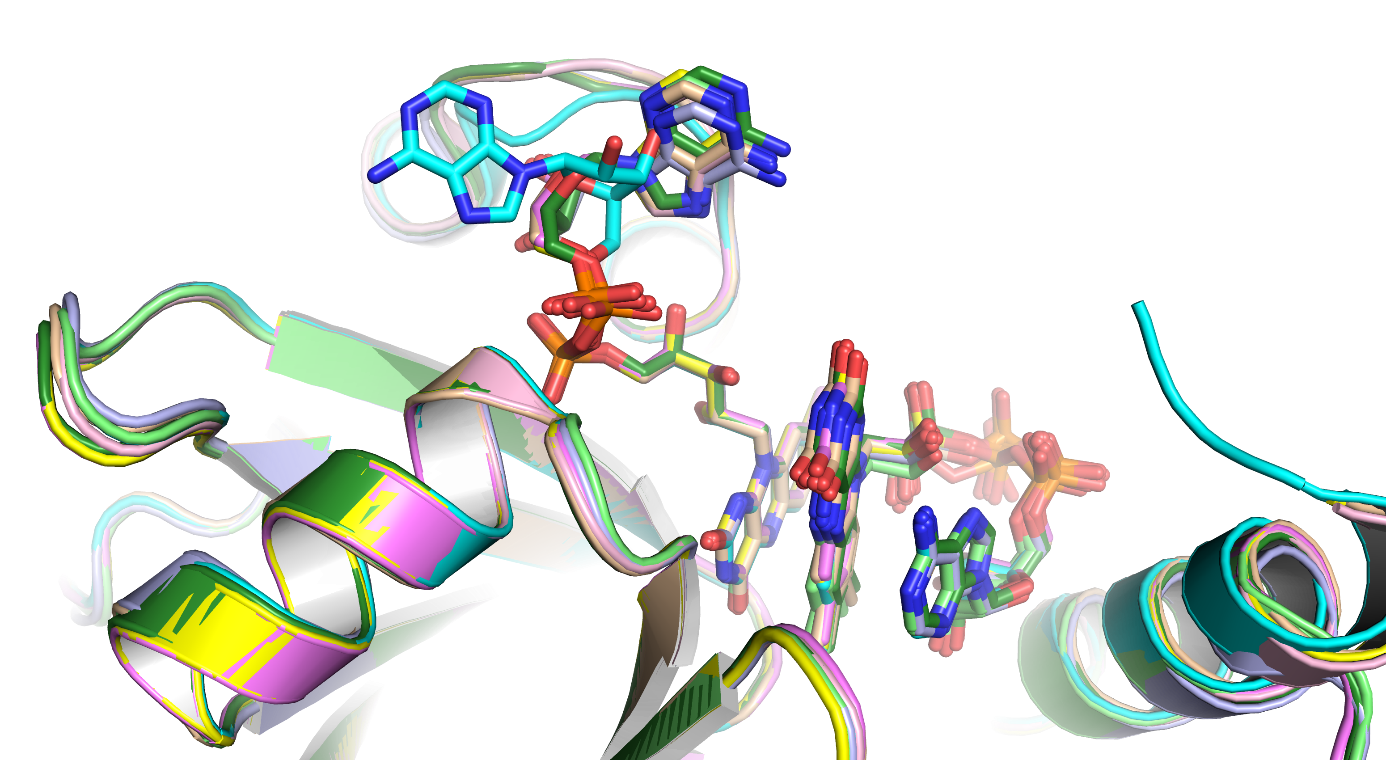
**

**Figure S5: Overlay of the eight crystallographically independent ThdF chains from crystal forms I and II (PDB 9fd4 and 9fd5)**. For the extended FAD, the FMN moiety and the phosphate from the AMP moiety are structurally almost invariant, while the adenosine shows substantial variation, especially in chain A of crystal form I (cyan carbon atoms).

**
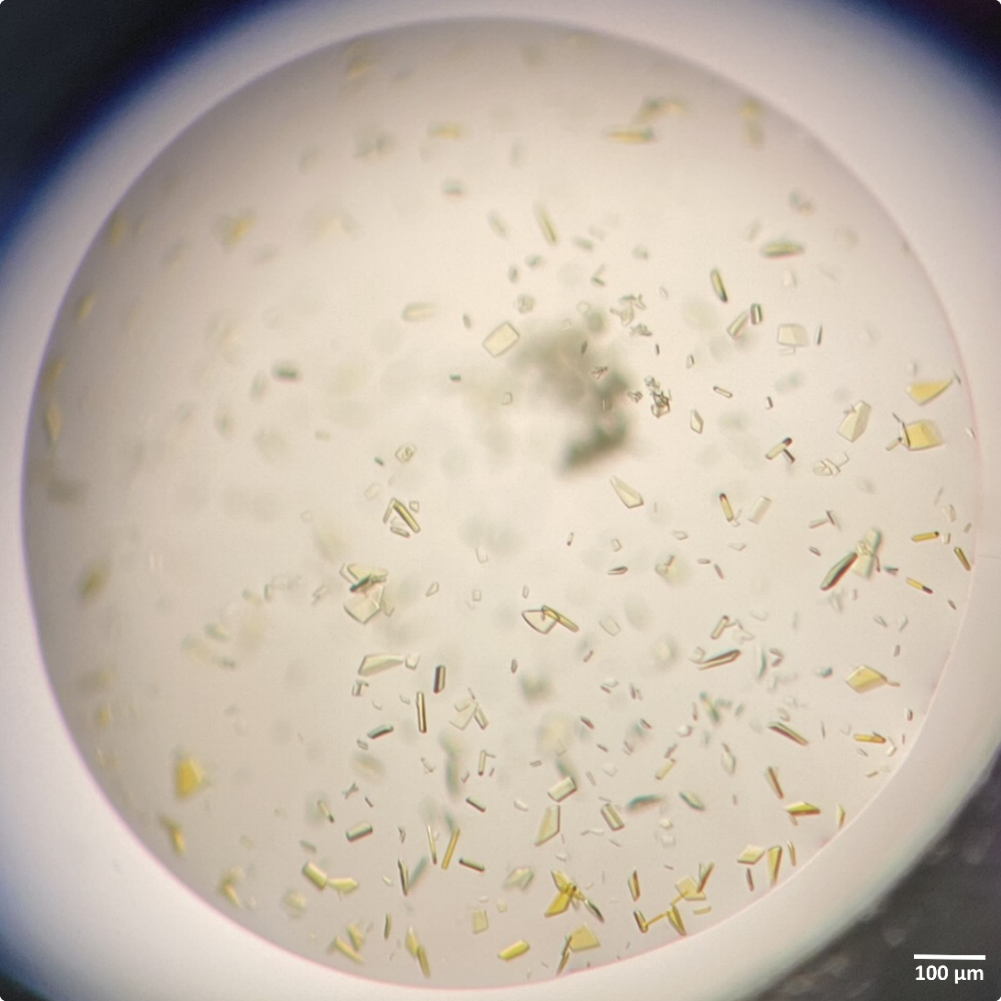
**

**Figure S6: Color change of ThdF crystals upon addition of dithionite.** ThdF crystals in condition D12 of JCSG++ (40 mM KH_2_PO_4_, 20 % (v/v) glycerol, 16 % (w/v) PEG 8000) immediately after adding NADH and dithionite. The loss of yellow color indicates a reduction of the bound FAD in the crystals. After a few seconds, all crystals appeared to be colorless.


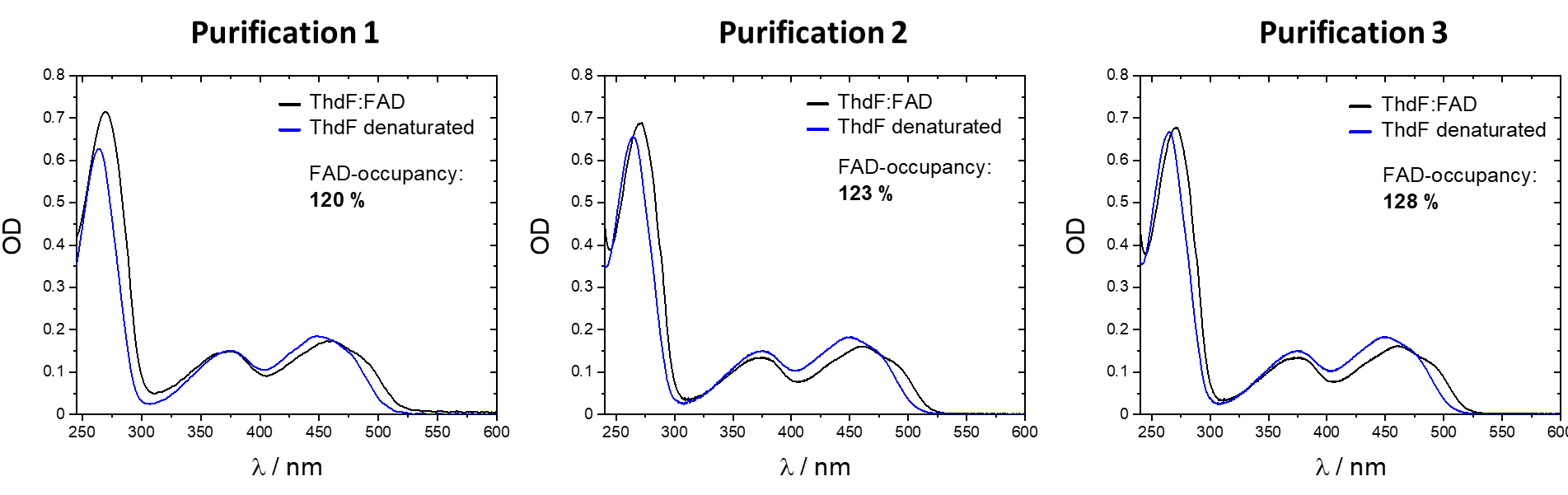


**Figure S7: UV-vis spectra of ThdF from three independent purifications.** FAD concentrations for accurate determination of FAD occupancy for ThdF were calculated from UV-vis spectra after removing the protein by boiling and centrifugation.

**
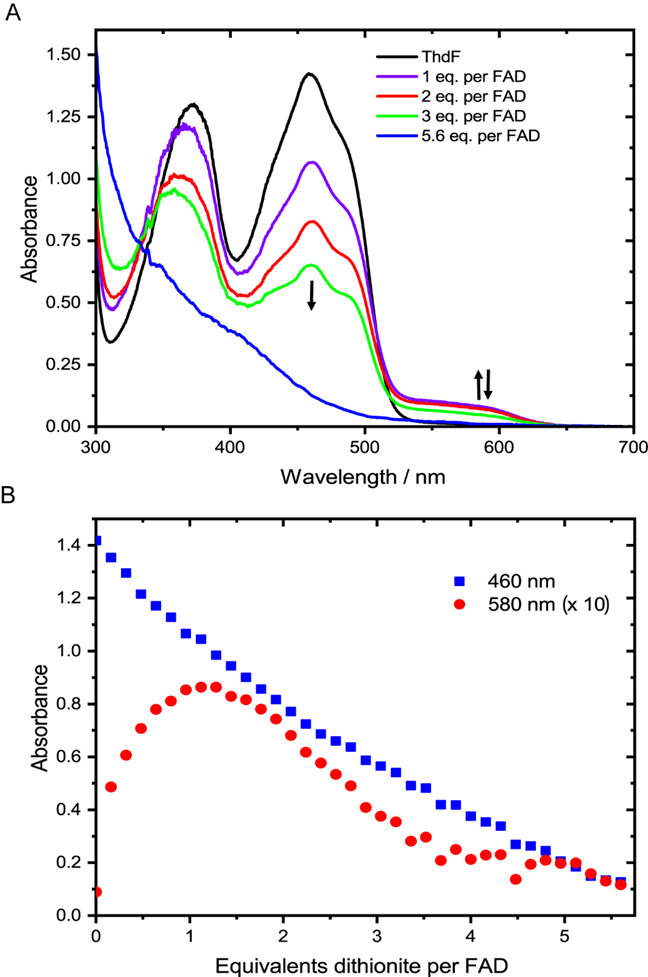
**

**Figure S8: Titration of ThdF with dithionite.** (**A**) The absorbance spectra at the specified points of the titration show that the absorbance of oxidized FAD decreased constantly. An FAD neutral radical was built up characterized by the absorbance between 540 and 620 nm, which decreased upon further addition of dithionite. The final spectrum showed a complete conversion to the fully reduced state of FAD. (**B**) The time course of the flavin absorbance at 460 and 580 nm as function of the addition of dithionite shows that the FAD neutral radical formed upon the addition of the first equivalent of dithionite. The maximal concentration of the FAD neutral radical was determined to 18 µM from the absorbance at 580 nm with ε_580_= 4800 M^‑1^cm^‑1^ (52). This concentration is equivalent to a conversion of 14% of the initial amount of oxidized FAD.


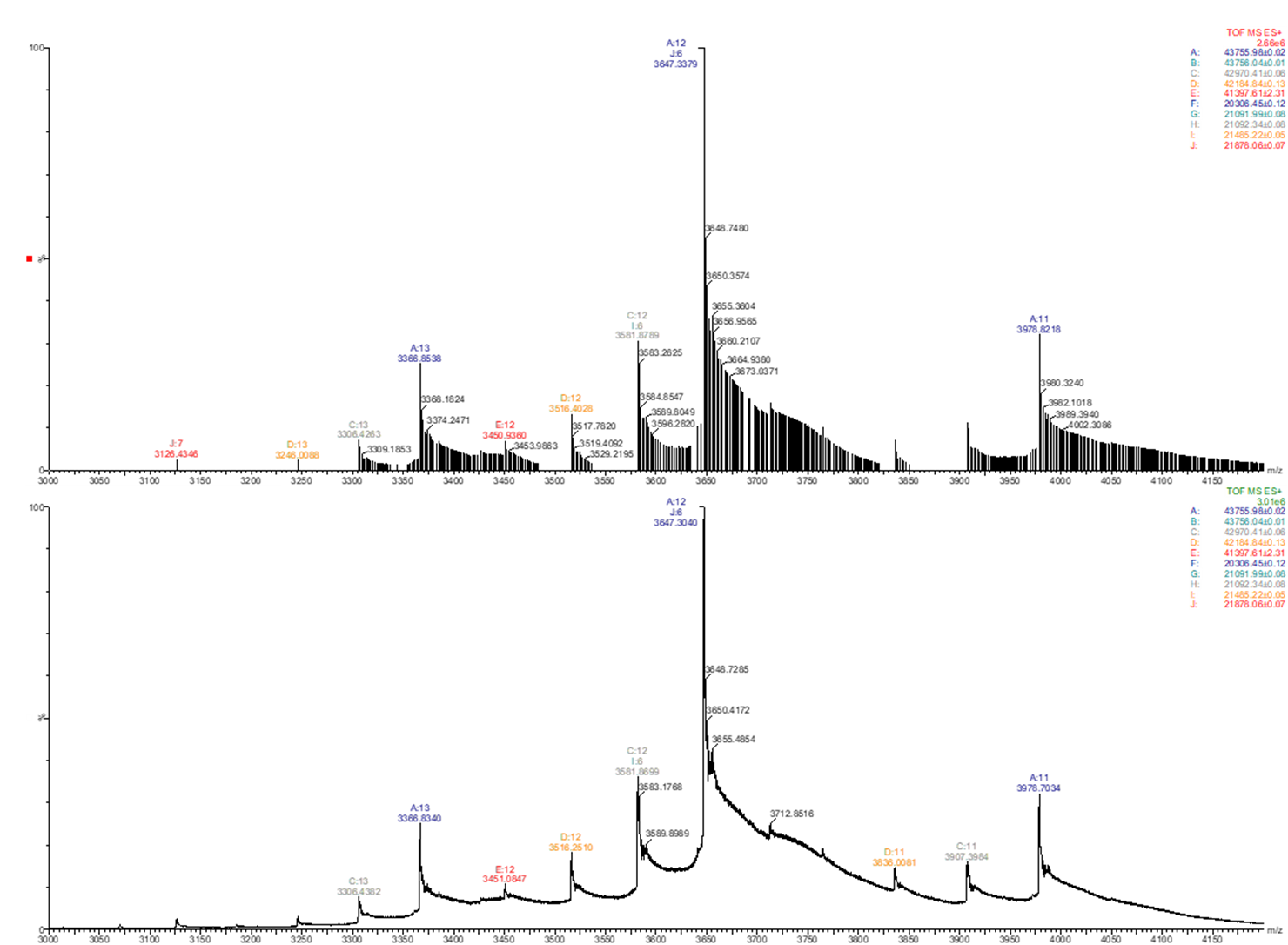


| **Series** | **m(exp)** | **m(theo)** | **Corresponding complex** |
| --- | --- | --- | --- |
| A/B | 43754.98 +/- 0.02 Da | 43756.04 Da | ThdF_2_FAD_4_ |
| C | 42970.41 +/- 0.06 Da | 42970.5 Da | ThdF_2_FAD_3_ |
| D | 42184.84 +/- 0.13 Da | 42184.9 Da | ThdF_2_FAD_2_ |
| E | 41397.61 +/- 2.31 Da | 41399.4 Da | ThdF_2_FAD_1_ |

**Figure S9: Mass spectra of ThdF obtained by native MS analysis.** Native mass spectrometry of ThdF in 50 mM ammonium acetate revealed several charge state series that can be assigned to ThdF dimers with different FAD occupancies. The lower panel shows the unprocessed data, the upper panel the MS data base line subtracted, smoothed, and centroided data. Masses of the protein:FAD complexes were determined using Transform.


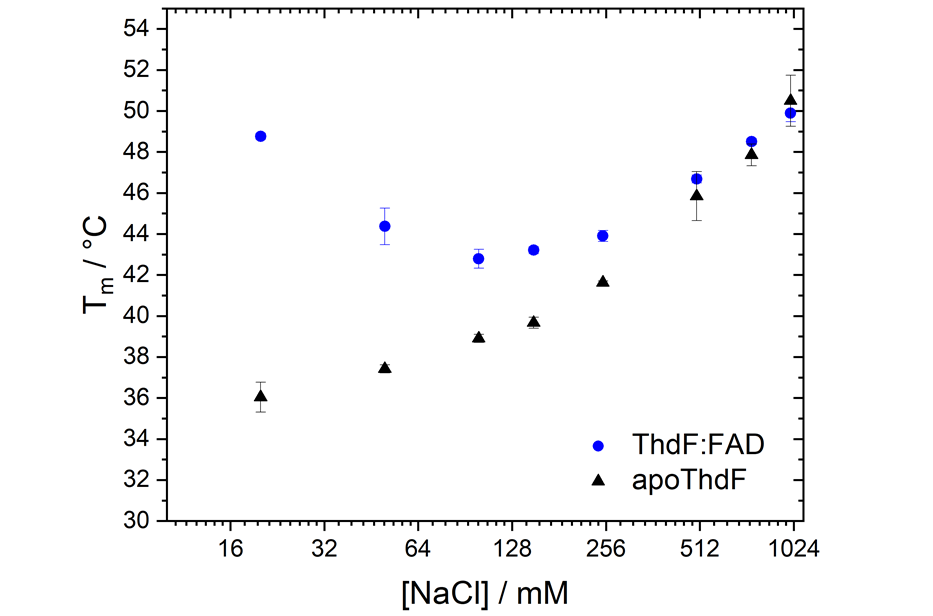


**Figure S10: DSF analysis of apoThdF and ThdF:FAD** **showing the melting temperature T_m_** **as a function of the salt concentration.** Each data point represents the mean and standard deviation of three technical replicates on one plate.


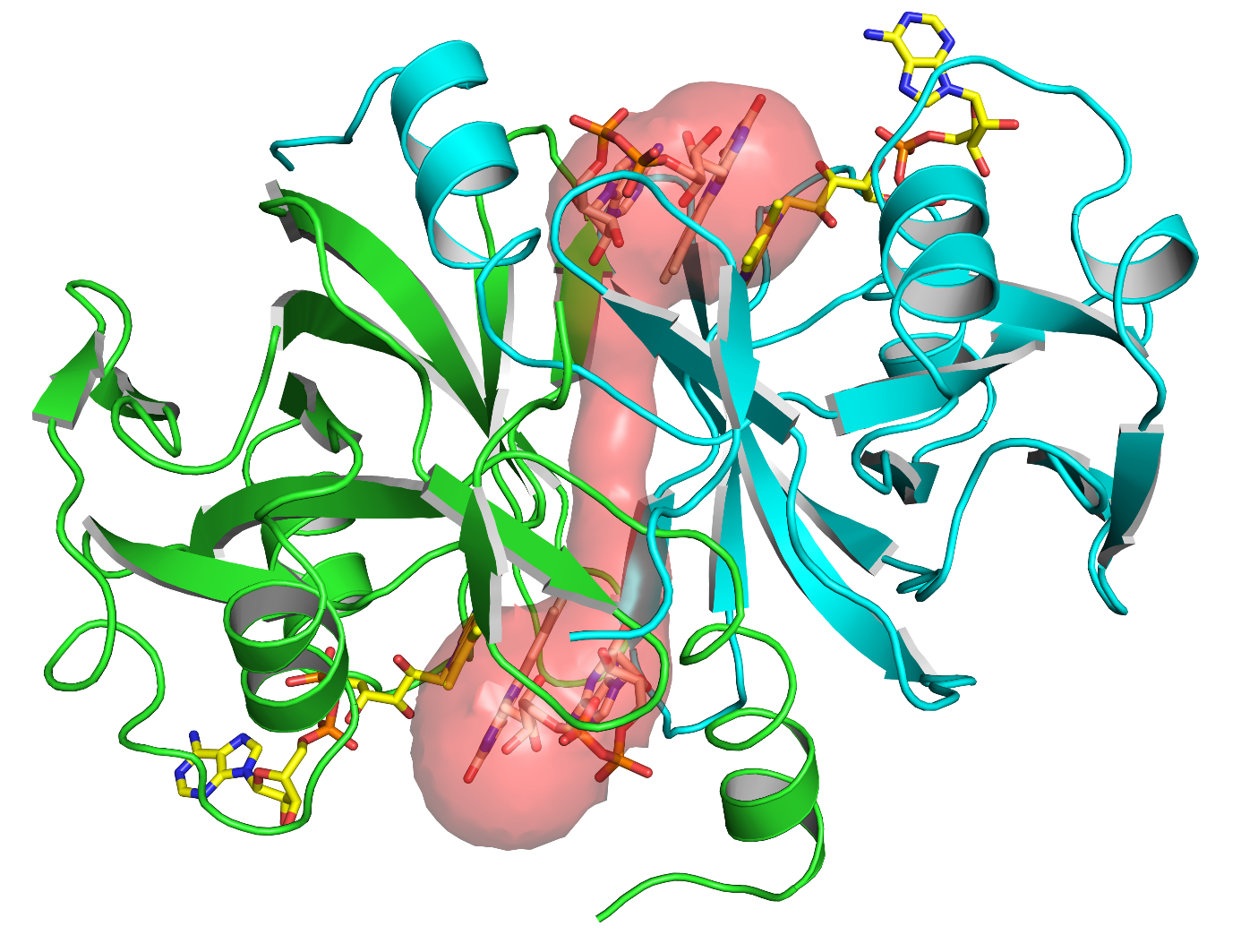


**Figure S11: Solvent channel at the ThdF dimer interface**. The tunnel connecting the two substrate binding sites was found by CAVER 3.0 (53, 54) with a probe radius of up to 1.61 Å.


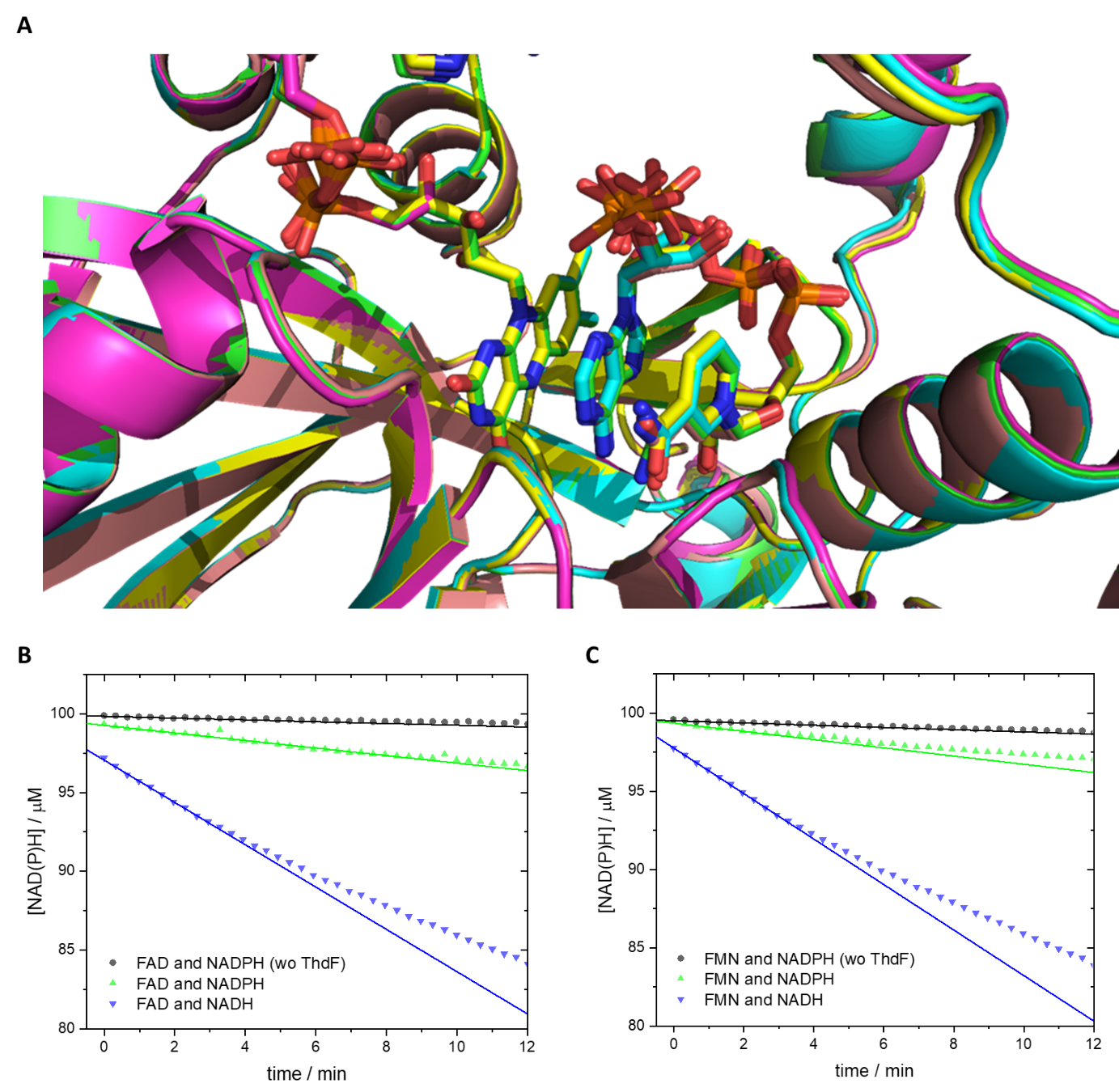


**Figure S12: An AlphaFold3 model provides a potential explanation why ThdF uses NADPH inefficiently.** (**A**) AlphaFold3 prediction of a ThdF dimer in complex with FAD and NADPH. The five top-scored structures are shown with differently colored carbon atoms. In all structures, the NADPH binds with its adenosine moiety close to the FAD, which should not allow a hydride transfer. (**B/C**). ThdF catalyzed flavin (**B**: FAD; **C**: FMN) reduction with NADH and NADPH. Oxidation of NAD(P)H was followed at 340 nm with 100 μM NAD(P)H, 10 μM flavin and 50 nM ThdF. While NADH is well accepted as a substrate by ThdF, the turnover of NADPH is only slightly higher than NADPH oxidation in the absence of the reductase.


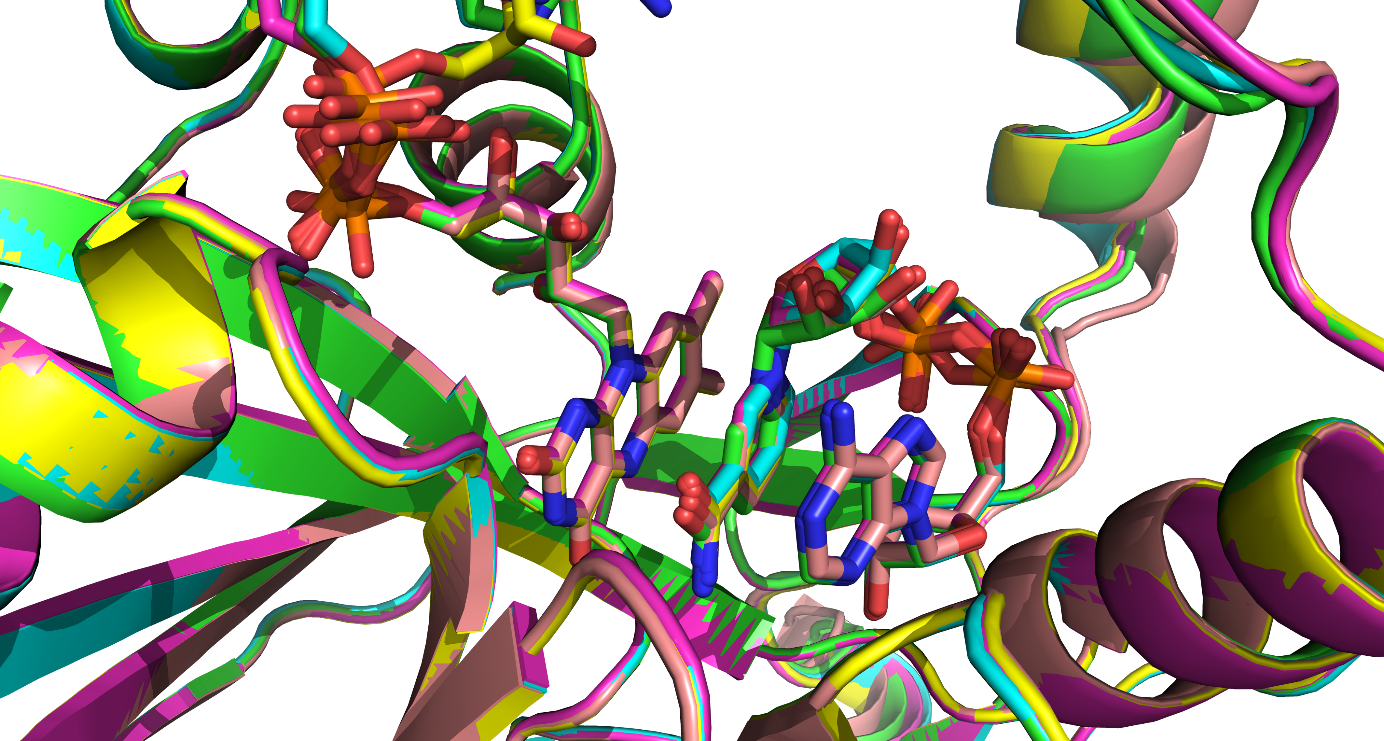


**Figure S13: AlphaFold3 prediction of a ThdF dimer in complex with NAD^+^ and FAD.** The five top-scored structures are shown with differently colored carbon atoms.


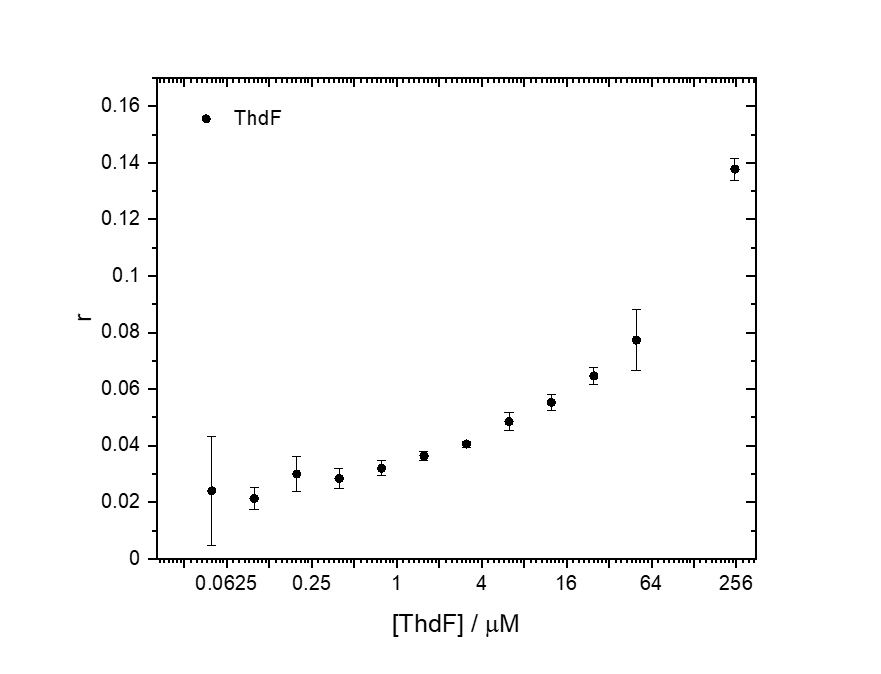


**Figure S14: FAD fluorescence anisotropy depending on ThdF concentration.**

**References**

33. Purich, D. L. (2010) *Enzyme Kinetics: Catalysis & Control*, *Elsevier*, *Amsterdam, The Netherlands*

45. Tickle, I. J., Flensburg, C., Keller, P., Paciorek, W., Sharff, A., Vonrhein, C., and Bricogne, G. (2018) Staraniso. *Cambridge, United Kingdom Glob. Phasing Ltd*

52. Wang, B., and Jorns, M. S. (1989) Reconstitution of Escherichia coli DNA Photolyase with Various Folate Derivatives. *Biochemistry*. **28**, 1148–1152

53. Chovancova, E., Pavelka, A., Benes, P., Strnad, O., Brezovsky, J., Kozlikova, B., Gora, A., Sustr, V., Klvana, M., Medek, P., Biedermannova, L., Sochor, J., and Damborsky, J. (2012) CAVER 3.0: A Tool for the Analysis of Transport Pathways in Dynamic Protein Structures. *PLoS Comput. Biol.* **8**, 23–30

54. Pavelka, A., Sebestova, E., Kozlikova, B., Brezovsky, J., Sochor, J., and Damborsky, J. (2016) CAVER: Algorithms for Analyzing Dynamics of Tunnels in Macromolecules. *IEEE/ACM Trans. Comput. Biol. Bioinforma.* **13**, 505–517
